# Water Stress Strengthens Mutualism Among Ants, Trees, and Scale Insects

**DOI:** 10.1371/journal.pbio.1001705

**Published:** 2013-11-05

**Authors:** Elizabeth G. Pringle, Erol Akçay, Ted K. Raab, Rodolfo Dirzo, Deborah M. Gordon

**Affiliations:** 1 Department of Biology, Stanford University, Stanford, California, United States of America; 2 Michigan Society of Fellows, University of Michigan, Ann Arbor, Michigan, United States of America; 3 Department of Ecology and Evolutionary Biology, University of Michigan, Ann Arbor, Michigan, United States of America; 4 School of Natural Resources and Environment, University of Michigan, Ann Arbor, Michigan, United States of America; 5 National Institute for Mathematical and Biological Synthesis, University of Tennessee, Knoxville, Tennessee, United States of America; 6 Department of Ecology and Evolutionary Biology, Princeton University, Princeton, New Jersey, United States of America; 7 Carnegie Institution for Science, Stanford, California, United States of America; Cornell University, United States of America

## Abstract

Abiotic environmental variables strongly affect the outcomes of species interactions. For example, mutualistic interactions between species are often stronger when resources are limited. The effect might be indirect: water stress on plants can lead to carbon stress, which could alter carbon-mediated plant mutualisms. In mutualistic ant–plant symbioses, plants host ant colonies that defend them against herbivores. Here we show that the partners' investments in a widespread ant–plant symbiosis increase with water stress across 26 sites along a Mesoamerican precipitation gradient. At lower precipitation levels, *Cordia alliodora* trees invest more carbon in *Azteca* ants via phloem-feeding scale insects that provide the ants with sugars, and the ants provide better defense of the carbon-producing leaves. Under water stress, the trees have smaller carbon pools. A model of the carbon trade-offs for the mutualistic partners shows that the observed strategies can arise from the carbon costs of rare but extreme events of herbivory in the rainy season. Thus, water limitation, together with the risk of herbivory, increases the strength of a carbon-based mutualism.

## Abstract 2

When water is scarce, trees invest in the moderate carbon cost of supporting defensive ants to avoid the potentially high carbon cost of extremities being eaten.

## Author Summary

The strength of ecological interactions, measured as the costs or benefits sustained by each species, depends on the environmental context in which the interaction occurs. Stressful environmental conditions should favor trading between species that can produce a given resource or service at the lowest cost. Mutualisms, in which both interacting species incur a net benefit, may thus strengthen under stressful conditions. Here we examine an ant–plant mutualism, in which plants provide food and housing for ants and ants defend plants against leaf-eating insects, along a four-fold annual precipitation gradient comprising tropical sites in Mexico and Central America. We show that the strength of the mutualism, in terms of carbon investment by plants and leaf defense by ants, increases as water availability decreases. Carbon shortages are more frequent where water is scarce and increase the risk that plants will die if all of their leaves are eaten by herbivores. Trees appear to invest more in ant defenders when water is scarce to insure themselves against extreme herbivory. Water availability thus indirectly determines the outcomes of this ant–plant mutualism, which suggests that the increasing frequency of extreme climate events in the tropics will have important ecological consequences.

## Introduction

Mutualistic species interactions contribute to multiple ecosystem functions by driving processes as diverse as dispersal, pollination, defense, and nutrient transfer. Environmental context frequently determines the levels of investments made by mutualists. For example, low-quality environments should amplify mutualism if the cost to each partner of obtaining a limiting resource independently is higher than that of receiving the resource from the mutualistic partner [1],[2]. The question then becomes: for a given mutualism, what determines environmental quality? In some cases, environmental quality can be defined simply by the availability of the resource being exchanged. For example, plant–mycorrhizal mutualisms are stronger when nitrogen and phosphorus are limiting [3]. In addition, fungal endophytes can confer drought resistance to plant hosts, promoting stronger mutualism under water stress [4]. However, there are few known cases of mutualism strength mediated by a limiting resource that is not directly involved in the mutualistic exchange.

Ant–plant interactions are classic examples of defense mutualisms: plants provide ants with food (and, in myrmecophytic plants, nesting space), and ants protect plants from herbivores [5]. Environmental context determines how much ants improve plant performance [6]–[8]. In particular, the abiotic environment, approximated by latitude, appears to affect the outcomes of ant–plant interactions in different communities [6],[8]. Abiotic effects are difficult to discern across communities, however, because such effects can be confounded with biotic macro-patterns, such as the latitudinal diversity gradient [7],[8]. The few previous studies of abiotic effects within particular ant–plant species pairs have suggested that water stress can mediate plant performance and investment in ants. In one study, ants benefited nectar-producing *Inga* plants only in sunny microhabitats, perhaps by preventing water stress caused by herbivorous thrips in high sun [9]. In another study, myrmecophytic *Acacia* plants produced more extrafloral nectar in the dry season, which resulted in higher ant activity and aggression against herbivores than in the rainy season [10]. Interestingly, this increase in nectar production and ant aggression in the dry season actually coincided with a lower risk of herbivory than that occurring in the rainy season. This suggests that plants may invest more in ant defense when they are water stressed because the costs of herbivory under water stress are particularly high, and leaves are harder to replace when resources are scarce [11].

**Figure 1:**
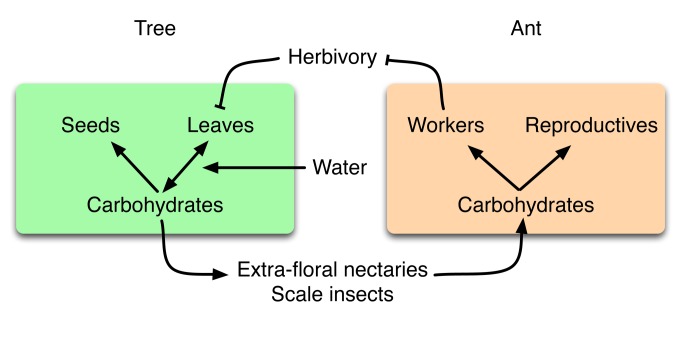
Schematic of relationships among water, carbon, and mutualistic carbohydrate allocation strategies. Trees and protective ants engage in an indirect exchange of carbon. Trees produce carbohydrates by photosynthesis and use them for growth, reproduction, and to support the scale insects that feed the defending ant colony. Water is necessary for trees to produce leaves and affects the efficiency with which leaves produce carbohydrates. Ants use carbohydrates to make sterile workers and reproductives. Workers defend the tree leaves from herbivory, and thereby protect the carbon source. Arrows denote positive effects; lines that end in Ts denote negative effects. In this study, we examine these relationships to test whether water stress leads to carbon stress and thereby increases the strength of the ant–plant mutualism.

Plant carbon limitation may be an important physiological mechanism mediating plant response to water stress [12],[13], but it is not clear how carbon limitation affects plant–animal mutualisms [14],[15]. Sustained water limitation causes plants to close stomata and reduce leaf area, which should increase the risk of carbon starvation [16],[17]. Such carbon stress would be exacerbated by leaf-area loss from herbivory (Figure 1). Because ants defend the leaves but represent a carbon cost to plants, plants under water stress should invest more in ant defenders if (1) water stress indeed leads to reduced carbon availability and (2) the carbon cost of maintaining ant colonies is lower than the cost of leaf herbivory.

An attractive system for studying these issues is provided by the myrmecophytic tree *Cordia alliodora* (Boraginaceae), which is found in tropical forests from Mexico to Argentina. In Mesoamerica, the most common ant symbiont is the *C. alliodora*–specialist *Azteca pittieri* (Formicidae: Dolichoderinae) [18]. Trees do not directly feed the ants but instead host phloem-feeding scale insects, whose sugar-rich excretions (known as “honeydew”) provide the ants with carbohydrates; trees must host scale insects to maintain ant colonies, and ants defend trees most effectively when densities of scale insects are high [19]. Thus, scales can be considered part of the tree's investment in defense. The trees also produce housing for ants in cavities at stem nodes (domatia), but the scale insects represent the primary cost to the trees for maintaining a defensive ant colony [20].

In seasonally dry tropical forests, where *C. alliodora* evolved and the mutualism is most common [18],[21], trees are deciduous and remain leafless throughout the annual dry season. Deciduous tropical trees face a particularly high risk of carbon starvation because they respire year-round at high temperatures (∼30°C) [17],[22],[23] but add to their carbon pools only during the rainy season. Tropical-rainfall seasonality alters the sizes of tree carbon pools within a site [24],[25], but to our knowledge, no study has documented intraspecific variation in the carbon pools of tropical trees at sites with different precipitation regimes. (One study looked for such variation but did not find it [26].) For *C. alliodora*, carbon stored from photosynthesis during the rainy season must be sufficient for dry-season respiration [17], leaf flush in the next rainy season [26], year-round maintenance of scale insects that feed defending ant colonies [19], and the tree's own reproduction [25]. Ant colonies must be maintained year-round to provide effective anti-herbivore defense in the rainy season (Figure S2).

In this study, we tested whether the strength of an ant–plant mutualism, measured as investment by trees in scale insects that feed ants and by ants in defense of leaves, is related to water availability across ∼2,300 km of Mesoamerica from Mexico to Costa Rica. We investigated the relationships among water, carbohydrates, and mutualistic traits across this gradient to test whether water stress leads to carbon stress, and whether carbon stress affects carbohydrate allocation strategies by the mutualists (Figure 1). We first asked whether precipitation was correlated with mutualistic investments by trees and ants. We then asked whether trees store more carbon, and experience a lower risk of carbon starvation, at sites where precipitation is higher and the growing (rainy) season is longer. Finally, to investigate when the cost of herbivory during the rainy season is high enough for trees to invest carbon in ant defense, we modeled carbon-allocation trade-offs for trees and ants in rainy seasons of different durations associated with different precipitation regimes.

## Results

**Figure 2:**
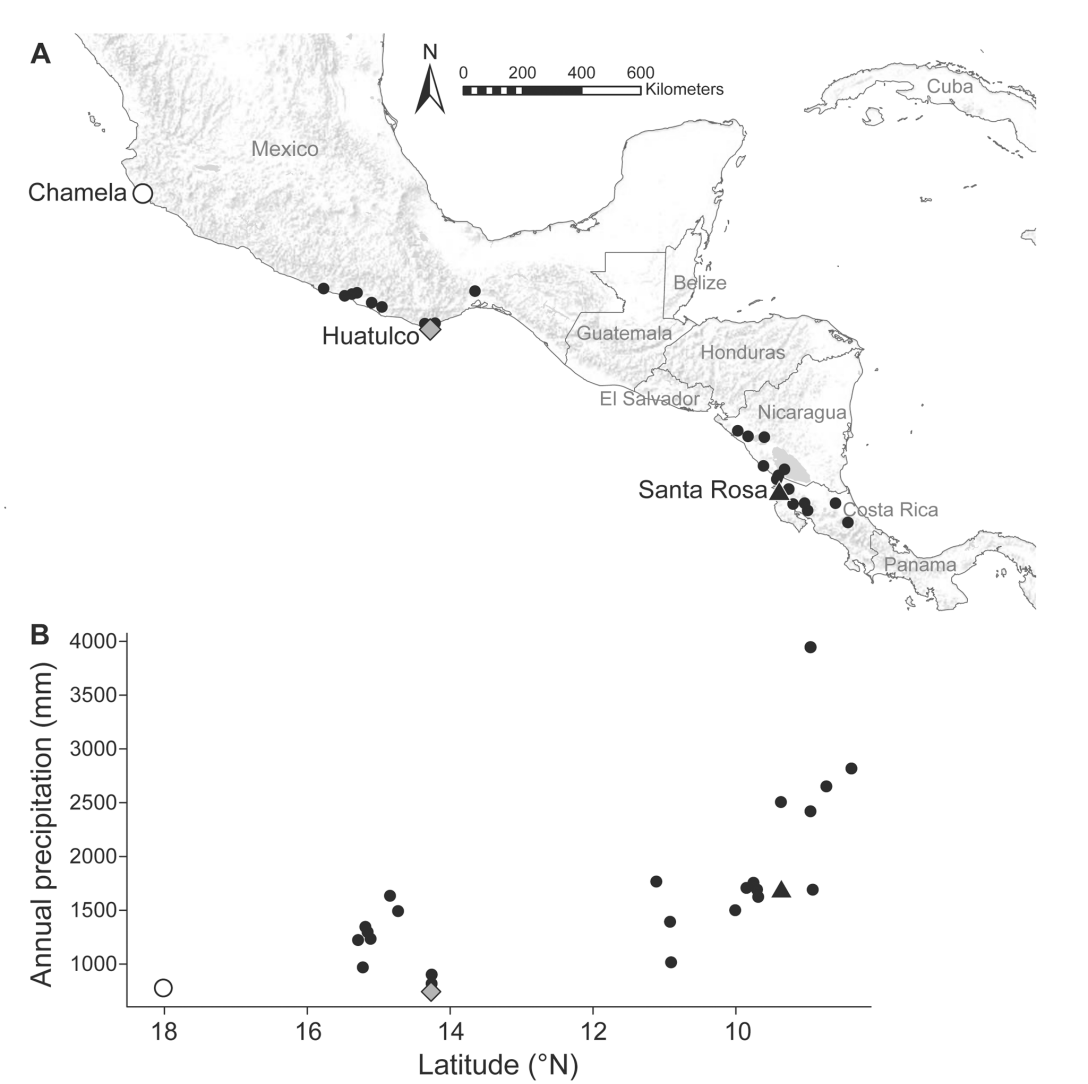
Study sites and precipitation gradient. (A) Map showing the 26 study sites and (B) their annual precipitation. Additional work was conducted at three of the sites, Chamela (white circle), Huatulco (gray diamond), and Santa Rosa (black triangle), that spanned the Mesoamerican dry-forest precipitation gradient.

To test whether water limitation leads to increased strength of a defense mutualism, and if so how, we studied the symbiosis between *C. alliodora* trees and *Azteca* ants in 26 Mesoamerican sites encompassing a four-fold precipitation gradient (Figure 2; Tables 1 and S1). In contrast to precipitation, temperature increases only weakly with latitude (*R^2^* = 0.09, slope = 0.15, *p*<0.0001; Table S2), and plant-available soil nutrients do not co-vary along the gradient (Table S3). We controlled for differences in light availability among sites in our analysis (Table S4). In the dry forests along the Pacific Mesoamerican coast, annual precipitation is positively correlated with the duration of the rainy season; dry seasons are longer at drier sites (Figures 2 and S1).

**Table 1:** Summary of the data and results used to support carbon-limitation hypothesis.

Data Class	Data	Variable	Factor	Correlation*	Evidence for main hypothesis**/against alternative hypothesis? (MH/AH)	Figure/Table
Environmental	Precipitation	Total annual (mm)	Latitude	**Negative**	MH	Figures 2, S1; Table S1
		Duration				
	Temperature	Annual mean (°C)		**Weakly positive**	AH	Table S2
	Soil nutrients	µg/g		None	AH	Table S3
	Light	% openness		**Positive**; Controlled	AH	Table S4
Organismal	Scale insects	Density per tree	Precipitation	**Negative**	MH	Figure 3A
	Ant workers	Density per tree		**Negative**	MH	Figure 3B
	Ant reproductives	Number per colony		None	MH	Table S5
	Ant defensive behavior	Patrolling ants (no.)		**Negative**	MH	Figure S5A
	Ant response	Latency (sec)		**Positive**	MH	Figures 3C, S5B, S4
	Ant exclusion	% leaf area		**Negative**	MH	Figure 3D
	Background herbivory	% leaf area		**Positive**	MH	Figures S9, S10
Organismal (Stoichiometric)	Tree NSCs	Total (mg/g)	Precipitation	Weakly positive	MH	Figures 4, S6; Tables S6, S7
		Starch (mg/g)		**Positive**	MH	
		Sucrose (mg/g)		**Negative**	MH	
	Tree allometry	Mass (g)		None	AH	Figure S7
		Leaf area (cm2)				
	Tree nutrients	Nitrogen to Phosphorus ratio		None	AH	Figure S8
	Ant isotopes	δ15N		None	AH	Figure S3A
	Ant nutrients	% Carbon, % Nitrogen		None	AH	Figure S3B,C
Theoretical	Optimality models	Tree carbon pool	Precipitation	Positive	MH	Figures 5, S10; Text S1
		Tree carbon invested in ants		Negative	MH	
		Ant carbon invested in workers		Negative	MH	

* Bold indicates significance at *p*<0.05 level.

** Mechanistic hypothesis illustrated in Figure 1 schematic.

**Figure 3:**
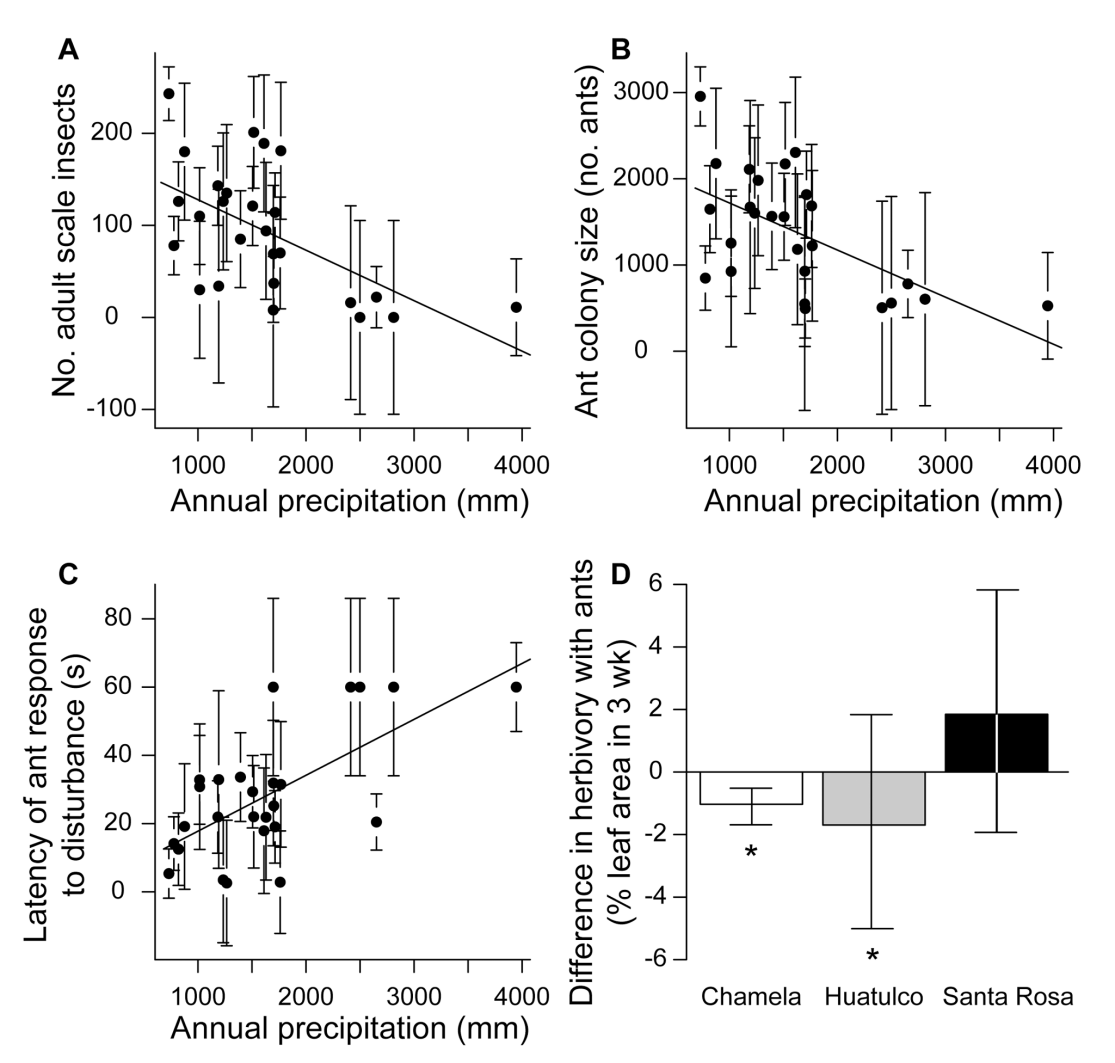
Mutualism strength increases as water availability decreases. (A) Relationship between annual precipitation of a site and the density of scale insects for all 26 sites (*R* ^2^ = 0.31, *F*_1,24_ = 12.15, *p*<0.002). (B) Relationship between annual precipitation of a site and ant colony size (density of worker ants) for all 26 sites (*R* ^2^ = 0.32, *F*_1,24_ = 12.95, *p*<0.002). (C) Relationship between annual precipitation of a site and the latency of ant response to a standardized disturbance (*R* ^2^ = 0.40, *F*_1,24_ = 17.87, *p*<0.0003). Error bars represent the square root of the total variance across sites divided by the sample size at each site. (D) Difference in percent leaf area eaten between when ants were present and when ants were experimentally excluded at three dry-forest sites along the annual-precipitation gradient from driest (Chamela) to wettest (Santa Rosa). Bars represent mean differences from matched-pair experiments within trees ± SE. Asterisks (*) indicate bars are significantly different from zero by one-sample matched-pair Wilcoxon tests (*p*<0.04); Chamela, *N* = 39; Huatulco, *N* = 27; Santa Rosa, *N* = 40.

The mutualism between trees and ants was stronger in drier sites than in wetter sites across the 26 surveyed locations from Mexico to Costa Rica (Figure 3). Trees supported more scale insects (Figure 3A), and ant colonies were correspondingly larger (Figure 3B) [19] in drier sites than in wetter sites. Colony size increased faster than the number of scale insects with water stress, such that ant colonies at a drier site (Chamela) tended fewer scale insects per worker ant than ants at a wetter site (Santa Rosa) (scale insects per ant = 0.25±0.02 in Chamela and 0.44±0.05 in Santa Rosa; *t* test, *p*<0.004). In contrast to the number of worker ants, colonies did not produce more female reproductives at drier sites (Table S5). Nitrogen isotopes indicated that ants fed at the same trophic level relative to host trees and scale insects at a drier and a wetter site (Figure S3A); ant tissues also contained equivalent percent carbon and nitrogen at different sites (Figure S3B–C). Ants defended trees more effectively in drier sites, responding more quickly to a disturbance to their host tree than in wetter sites (Figures 3C and S4). In ant-exclusion experiments conducted at three sites encompassing the extremes of the precipitation gradient, herbivory was significantly lower on leaves with ants than on leaves without ants in two of the driest sites (∼774 mm-rain/y) but not in a wetter site (∼1702 mm-rain/y) (Figure 3D). Behavioral assays of activity and responsiveness indicated that ants were more likely to find herbivores and chase them from trees in drier sites than in wetter sites (Figure S5).

**Figure 4:**
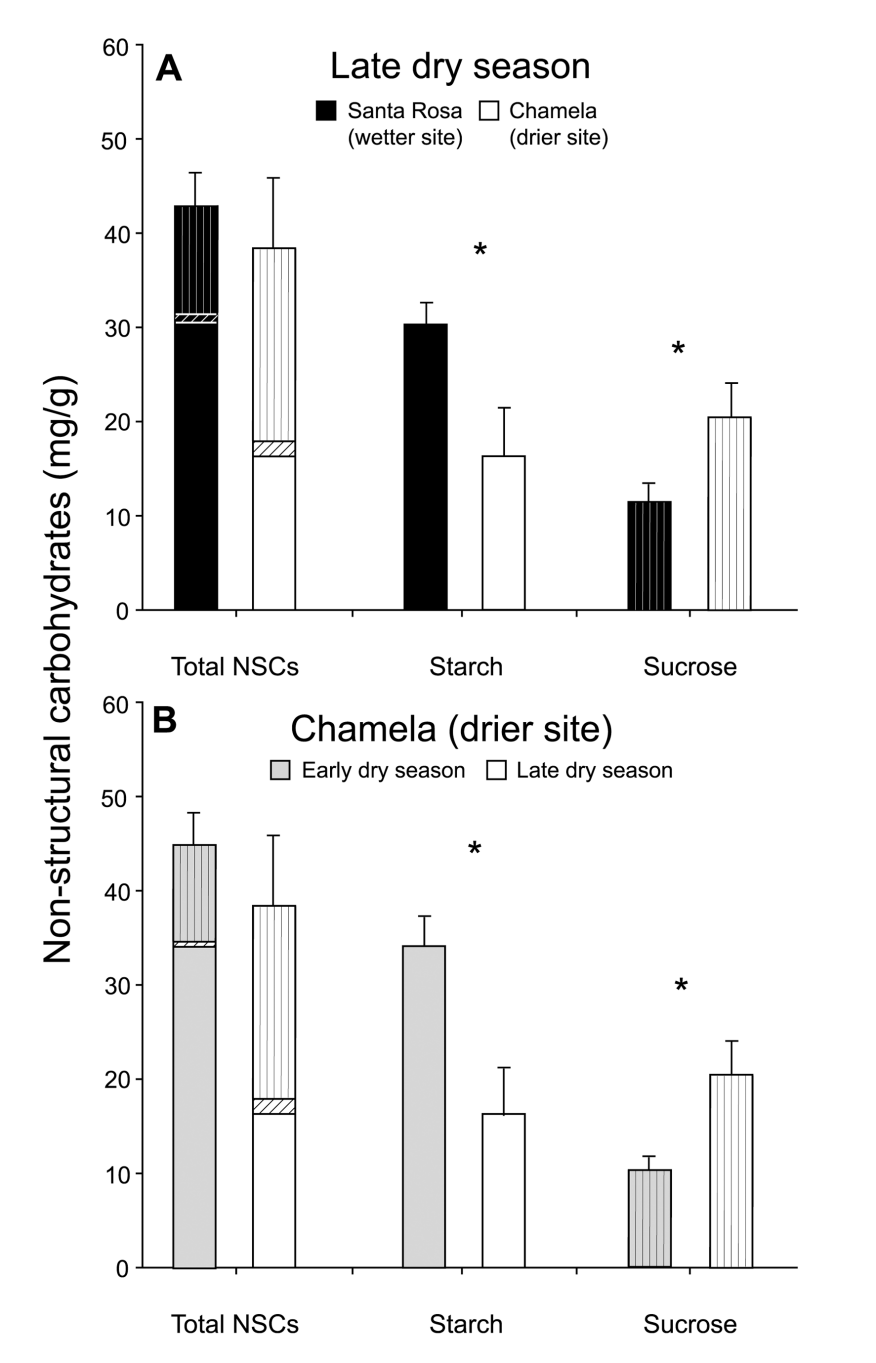
Tree NSC pools and water stress. Total NSCs, starch and sucrose pools in main stems in (A) the late dry season (April 2009) at a wetter site (Santa Rosa; black bars) and a drier site (Chamela; white bars) and (B) the early dry season (October 2009; gray bars) and the late dry season (April 2009; white bars) at the drier site. Bars indicate means, and error bars indicate SE; values are based on plant dry weights. Starch bars are open, glucose (free sugars) bars have diagonal lines, and sucrose bars have vertical lines. Asterisks (*) indicate *p*<0.05 by two-tailed *t* tests.

To test whether trees experiencing drier conditions retained smaller carbon pools, we measured total nonstructural carbohydrates (NSCs) in *C. alliodora* trees in two sites that differed two-fold in annual precipitation (Figure 2; Figure S1). Main stems and roots were the primary NSC storage tissues at both sites (Table S6). We found that, as predicted, tree starch pools in main stems were significantly smaller in the drier site than in the wetter site at the end of the dry season (Figure 4A; Table S6). Additionally, at the end of the dry season, main stems contained a higher ratio of sucrose to starch in the drier site than in the wetter site, which reduced the difference between sites in total NSCs (Figure 4A; Figure S6; Table S6). The conversion of starch to sucrose maintains osmotic balance during severe water stress and is the first stage of carbon-pool depletion [23], which increases the probability of carbon starvation [13]. Between-site differences were mirrored by seasonal differences within the drier site, where starch reserves were significantly depleted over the course of the dry season and sucrose∶starch ratios increased concomitantly (Figure 4B). Differences in carbon pools were attributable to differences between sites in water limitation, not to differences in leaf area, temperature, nutrients, or light (Figures S7 and S8; Tables S2, S3, S4).

Tree NSC pools at both sites exhibited strong seasonal patterns. Trees exhibited the lowest levels of NSCs in the early rainy season shortly after flushing new leaves (i.e., stored carbohydrates are invested in new growth) and the highest levels of NSCs in the early dry season when growth slows and leaves are still present [25]. Shorter growing seasons at drier sites than at wetter sites (Figure S1) led to larger NSC pools in the drier site, where trees had stopped growing, than in the wetter site, where trees continued to grow, in late October 2009 (Figure S6). In the week preceding this tissue collection, it rained 6 mm in the drier site and 40.2 mm in the wetter site; in the month following tissue collection, it rained 0 mm in the drier site and 314.9 mm in the wetter site. The time of tissue collection thus represented the early dry season in the drier site but the late rainy season in the wetter site. Twig NSCs mirrored patterns in main stems (Figure S6), but twigs varied less in the dry season and more in the late rainy/early dry season than main stems (Tables S6 and S7).

For trees at drier sites to invest more carbon in ant defenders even when they face a greater risk of carbon starvation, the cost of leaf herbivory must be higher than the cost of ant defenders. To investigate when this is the case, we modeled the indirect carbon trading between trees and ants, via scale insects and leaf defense (Figure 1), in rainy seasons of different length. We compared two models, an “insurance” model in which ants protect trees from rare and extreme events of herbivory, and a “chronic herbivory” model, in which ants protect trees from sustained herbivory throughout each rainy season. In the insurance model, extreme defoliation forces the tree to produce new foliage, which costs the tree time for photosynthesis, whereas in the chronic-herbivory model, herbivory reduces the equilibrium leaf area for the duration of the rainy season. In both models, we concentrate on the cost of sustaining an ant colony during the rainy season, when colonies grow and reproduce, rather than during the dry season, when they do not (see Materials and Methods; Text S1).

**Figure 5:**
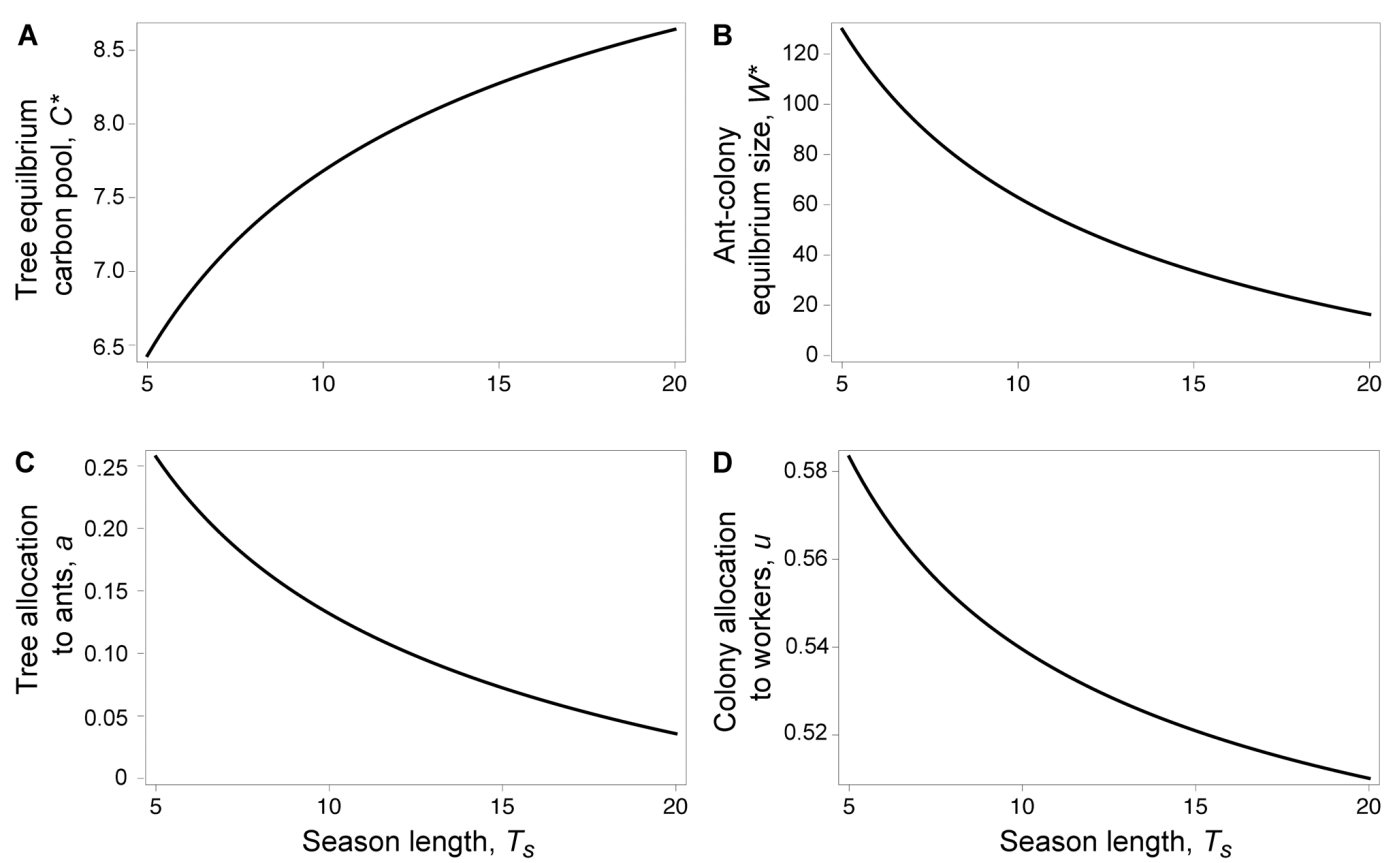
Insurance model of indirect carbon trading. Equilibrium (A) tree carbon pools, C^*^, and (B) ant colony size, W^*^, as allocated in each mutualist's evolutionary stable strategy as a function of growing-season (rainy-season) duration. (C) Tree carbohydrate investment in ants, *a*, and (D) ant investment in colony growth, *u*, as a function of the season length T_s_ in the evolutionary stable strategy, while keeping the time a tree needs to replace its leaf area, *τ*, constant. Parameter values are: *h* = 0.1, *L_max_* = 10, *τ* = 5, *μ* = 0.01, *k* = 10, and *q_0_* = 1.

The insurance model predicted, as we observed, that shorter rainy seasons led to stronger mutualism by means of smaller tree carbon pools (Figure 5A), more carbohydrate allocated to ants by trees (Figure 5B), and larger ant colonies (Figure 5C) that use more of their tree-provided carbon to produce defending ant workers (Figure 5D). Moreover, this model is consistent with recent demonstrations that ants are costly to plants at short timescales [27],[28] because the relevant selective events shaping the mutualism (i.e., complete defoliation) are predicted to be catastrophic and rare (Figure S9). In the insurance model, mutualism strength can increase with water limitation even if the probability of defoliation decreases (Figures S9 and S10). The chronic herbivory model predicted, contrary to what we observed, that rainy-season length alone had no effect on mutualism strength, and that mutualism strength should decrease with water limitation if the rate of defoliation also decreases (Text S1; Figure S11). The difference in the predictions of the two models can be explained by the timescale of herbivory relative to rainy-season length. In the chronic herbivory model, ant defense benefits the tree more when the rainy season is longer, because the leaf area saved from herbivory has longer to produce more total carbon. In contrast, in the insurance model, ant defense benefits the tree more when the rainy season is shorter, because the time necessary for leaf replacement following complete defoliation is a greater proportion of the total rainy season. Therefore, according to the insurance model, trees exposed to the risk of extreme herbivory should invest more in their ant defenders when there is less time each year to produce vital carbon.

## Discussion

Mutualistic interactions should be stronger under resource-poor conditions [1], but empirical support for this prediction has been restricted to mutualisms whose currency of exchange is the limiting resource itself. In this study, we have shown that the strength of an ant–plant mutualism increases with water stress across 26 sites spanning 2,300 km of Mesoamerican seasonal forests. These data are consistent with previous studies of other ant–plant mutualisms, suggesting a positive link between water stress and mutualism strength [9],[10]. Our results indicate both that these changes result from increased carbon stress on plants in drier sites and that such plants invest more carbon in their ant defenders when faced with the risk of extreme herbivory. We suggest that an abiotic variable, precipitation, and a biotic variable, herbivory, together drive macroscale patterns in the strength of a plant–animal mutualism by means of indirect effects on plant carbon availability.

Extreme drought causes large-scale tree mortality in the tropics via physiological mechanisms that depend on feedback between carbon shortages and hydraulic failure [29],[30]. The seasonal trends that we detected in *C. alliodora* NSC pools are comparable to those reported from a dry forest in Panama [25]. However, in contrast to the previous study, we detected significant reductions in starch and higher sucrose∶starch ratios in main stems in the late dry season at our drier site (where annual precipitation is ∼1,000 mm lower than in Panama). This suggests that even in the absence of drought (precipitation in 2008 preceding our late-dry-season measurements was higher than average at the Chamela site; Figure S1A), trees at drier sites are water stressed during the dry season, which reduces starch pools and increases the risk of carbon starvation. Years of extreme drought may thus create intense selective pressure for trees to produce sufficient carbon during the rainy season; such pressure should also lead to increased investment in leaf defense. A study of variation in hydraulic traits among three populations of *C. alliodora* found that trees from drier sites were significantly more resistant to drought-induced embolism than trees from wetter sites [31]. Resistance to embolism is often a genetic trait [31], so *C. alliodora* hydraulic traits may have adapted to local water availability, despite high gene flow across the tree's range [32]. An interesting and unanswered question is whether and how selection may also act on tree carbon-allocation traits.

How do trees alter their carbon investment in ant defenders in this system? Colonies of *A. pittieri* ants are not limited by nesting space [19], and trees do not produce food rewards directly. We hypothesize that trees can indirectly alter their investment by changing phloem chemistry, which affects scale-insect success and attractiveness to ants (e.g., [33],). Direct evidence for such a plant strategy in any system is scarce, but it is almost certainly true that myrmecophytic plants have evolved defenses against scale insects, given that nearly 90% of symbiotic ant–plant systems also contain ant-tended scales [35]. Indeed, the ubiquity of these associations suggests that scales play a key role in the evolution of ant–plant symbioses, perhaps by providing ants with nutrients that are scarce in the plants themselves [36]. Phloem may contain few of the secondary metabolites typically associated with plant defense [37], but studies of phloem chemistry are extremely limited [36],[37], and primary metabolites should be particularly effective in defense against sedentary insects, such as scales [33]. The quality of honeydew that ants receive is closely related to phloem quality [37], so ants should be able to detect and respond to changes in phloem content. Previous work in other systems has shown that hemipterans respond to phloem nutrient content [33] and that plant genotype determines honeydew attractiveness to ants [34]. Thus, although ants may directly control the number of scale insects [19], decisions about how many scales to tend could be driven by the quality of honeydew that the ants receive. Future work is needed to clarify the roles of constitutive and induced defenses in plant phloem, and of the possible effects on ant behavior.

Our finding that ants are better defenders at drier sites (Figure 3D; Figure S5) suggests positive mutualistic feedback from the ants in response to an increased supply of tree carbon at drier sites. Although ant colonies tended more scales at drier sites, ants did not increase their own fitness at the tree's expense: colonies produced more sterile workers that defend plants, not more female reproductives (Figure 3B; Table S5). These changes in ant life-history strategies and behavior could be ecological, or ants may genetically adapt to local conditions. Local adaptation is facilitated by restricted gene flow, and unlike their host trees, *A. pittieri* ants exhibit pronounced genetic structure in Mesoamerica [18]. Consistent with the possibility that genetic lineages of *A. pittieri* are locally adapted, northern and southern *A. pittieri* lineages do not overlap in their precipitation niches, and genetic divergence does not correspond simply to isolation by distance [18]. The next step will be to ask whether ant defensive behaviors exhibit phylogenetic patterns consistent with local adaptation of mutualistic strategy.

We have focused here on the apparent role of carbon in regulating the mutualistic exchange between trees and ants (Figure 1). Two alternative hypotheses to explain the negative relationship between scale-insect abundance and annual precipitation deserve consideration. First, water availability could have independent, direct effects on both plants and ants. If ants are directly water stressed, they might tend more scales to obtain water at drier sites. Our results indicate that this is unlikely, because ant-colony size and the number of scale insects increased concomitantly with water stress, such that ants at drier sites actually tended fewer scale insects per ant (see Results). Second, a currency besides carbon could regulate ant–plant mutualistic exchange. For example, ants can provide plants with nitrogen and other nutrients (e.g., [38]), and arboreal ants may themselves be nitrogen-limited [39]. We have suggested previously that *A. pittieri* ants obtain their nitrogen primarily from dead insects and guano [19], sources that could vary substantially among sites. Arguing against a key role for nutrients other than carbon, however, is that our data have consistently shown nonsignificant differences in nutrient content of both plants and ants between sites (Figures S3 and S8; Table S3). Nitrogen-isotope data showed that ants at different sites were trophically indistinguishable (Figure S3). The ^15^N-to-^14^N isotope ratio was higher at our drier site than at our wetter site, a difference that may be driven either by precipitation regime [40] or by other factors, such as soil sources and associated microbial communities [41]. The result relevant to our study, however, is that the relative increase in 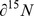 from plants to scale insects to worker ants was constant among sites (Figure S3), which indicates that differential nitrogen limitation does not drive geographic variation in ant behavior in this system.

Recent research has indicated that ant colonies require much more carbohydrate than protein for colony growth [42],[43]. An open question is whether differences between sites in scale-insect diversity, and consequent differences in overall honeydew composition [44], can alter ant behavior. *Cryptostigma* spp. and *Paraputo* spp. are the most abundant scale-insect genera tended by *A. pittieri* throughout Mesoamerica [19], but honeydew chemistry can be species-specific [44], and this deserves attention in future studies. Whatever the chemical differences in honeydew between sites, however, ants received significantly more carbohydrates at drier sites than at wetter sites (Figure 3A). Our model suggests a mechanism whereby carbon-stressed trees accrue a fitness advantage by this increased carbon investment in ant defenders.

According to our model, bet-hedging [45] by trees subjected to variation in growing-season length could drive the evolution of differences in carbon investment in this mutualism. Rare but extreme events should be particularly important selective agents in long-lived organisms [46]. Our results indicate that infrequent and extreme herbivory, as opposed to chronic, low-level herbivory, best explains the evolution of tree and ant mutualistic strategies in different precipitation regimes. In drier habitats, trees sustain the moderate cost of the symbiosis with ants and scales to avoid the potentially high cost of extreme herbivory. The importance of extreme herbivory to the evolution of symbiotic ant–plant mutualisms was previously proposed in an African system where elephants occasionally tear down entire trees [28]. In contrast, insects are the primary herbivores in our system. Outbreaks of herbivorous insects are better documented in temperate forests than in tropical ones, but such events are also important in the tropics [47]. Indeed, although relatively low herbivory of *C. alliodora* is typical (Figure S9), we have also observed occasional complete defoliation of some trees at our study sites over the past six years. We propose that although extreme herbivore outbreaks are rare, such events will occur at least once within the lifetime of a long-lived tree [46], such as *C. alliodora* [48]. Further long-term studies of herbivory in the tropics are necessary to confirm this hypothesis.

Overall, our work suggests that the drought-induced risk of carbon starvation in plants [12],[16],[17] influences the ecological outcomes of an ant–plant mutualism. Because carbon is the primary currency of plant mutualistic exchange, carbon limitation may be an important physiological mechanism mediating plant mutualistic strategies in many systems [14],[15]. Biotic extremes that operate synergistically with abiotic extremes can produce crucial selective events [46], and herbivore outbreaks may often coincide with extreme drought [47] to affect plant trait evolution. It is significant that the key abiotic variable that alters interactions in this system, precipitation, is a climatic one that is likely to change dramatically over the coming years. As extreme climatic events increase in frequency with climate change [49], ecological and evolutionary responses of reproductive individuals to physiological stress will drive changes in the outcomes of species interactions and ecosystem function.

## Materials and Methods

### Climate Data

Climate data for the 26 sites were extracted from 1-km resolution WorldClim datasets [50]. Additional temperature and rainfall data were collected in Chamela and Santa Rosa at biological stations associated with each site. Rainfall and temperature data for Huatulco were consolidated from incomplete datasets for the time period 1979–2009 from weather stations in three nearby towns, Santa María Huatulco, Puerto Ángel, and Pochutla.

### Soil Nutrients

Soil samples were collected 1 m from *C. alliodora* trees at depths of 0–5 cm and 15–20 cm. Field moist soil samples were sieved (2-mm sieve) and extracted in 2 M KCl for ammonium (NH_4_^+^) and nitrate (NO_3_^−^) assays [51]. Soil phosphate (PO_4_^3−^) was extracted using resin bags [52]. Extractions were analyzed on a discrete analyzer (Westco SmartChem 200). Soils were also analyzed for organic carbon by comparing ignition loss of 5 g of wet soil, dried at 60°C for 24 h, and combusted in a muffle furnace heated to 550°C for 2 h [53].

### Tree Growth and Canopy Openness

Basal diameter and diameter at breast height (DBH) were measured for trees in July and August 2007, and again in June and July 2010. To compare canopy openness, hemispherical photographs were taken with a fisheye lens (Sigma 8 mm F3.5 EX DG on a Canon EOS-1 Mark II Digital camera) at 1 m height in three 0.5-ha tree plots. Plots were divided into 10×10 m quadrants, and photographs were taken at each quadrant intersection (*N* = 35 photographs per plot). Photographs were analyzed in Gap Light Analyzer v. 2.0 [54].

### Mutualistic Investment

Ants and scale insects were counted and ant-exclusion experiments were conducted as described previously [19]. Ant colonies reproduce continually during the rainy season, and adult female reproductive alates were counted ∼1 mo into the rainy season at each site, respectively. Ant-exclusion experiments were conducted in the early rainy season in Chamela and Santa Rosa in 2008 and in Huatulco in 2009. A test for latency of ant response to a disturbance was conducted by hitting trees, using a standardized intensity, and timing ant response. In this test, EGP hit a tree with a hammer three times at breast height. The time was recorded at which ants were seen to leave domatia and patrol the plant more than prior to the test. We looked for a response for a maximum of 30 s. If there was no ant response in the first 30 s, EGP repeated the hammer test and waited up to 30 s. If there was no measurable ant response in that time period, latency to respond was recorded as 60 s.

### Ant Stable Isotopes and Elemental Composition

Ant workers were collected in the field and transferred to the freezer within 1 h. Frozen samples were then dried at 60°C for ≥48 h. The gaster of each worker was removed, and the head and alitrunks of ≥20 workers were ground together with a mortar and pestle. These samples were then analyzed for ∂^15^N, %C, and %N at the UC Davis Stable Isotope Facility.

### Tree NSCs

Tissues were collected from *C. alliodora* trees 2 to 15 m in height with canopies exposed to sunlight. We collected (1) stem tissue from an area of ∼3×4 cm located at breast height after removing bark, (2) coarse roots ≥0.5 cm in diameter, (3) three terminal twigs 0.2–1.2 cm in diameter, (4) three leaves in full sun, and (5) 20–50 mature, undamaged seeds. Stem and twig tissues were collected from six trees in Chamela and Santa Rosa in early rainy season (June–July 2008), late dry season (April 2009), and late rainy/early dry season (October 2009). All collections took place in the morning (8:00–11:00 h). All samples were transferred within 1 h to a drying oven at 60°C where they were dried for ≥48 h. The three branch and leaf samples for each tree were milled separately, carbohydrates were quantified separately, and results were averaged within individual trees prior to analysis. Starch was extracted with 90% dimethyl sulfoxide (DMSO) for 18–19 h in a 55°C water bath. Samples were then diluted to 20% DMSO and hydrolyzed with amyloglucosidase and β-amylase for 2 h at 37°C [55]. Free glucose and sucrose were extracted with 80% ethanol for 1 h at 35°C. Samples were then dried with nitrogen gas and re-diluted with one part chloroform to two parts water. Glucose samples were used directly from the clear water phase, and sucrose samples were hydrolyzed with invertase for 1 h at 55°C. Total glucose was then assayed for each sample using 10 units of peroxidase glucose-oxidase enzymes (Sigma Aldrich Inc.), 25 µM 3-(dimethylamino)benzoic acid, and 0.5 µM 3-methyl-2benzothiazolinone hydrazone [56]. Absorbance was measured on a Beckman-Coulter DU640 UV/Vis spectrophotometer at 595 nm wavelength [56].

### Tree Allometry and Nutrients

Tree above-ground dry biomass was determined for six trees at the sites Chamela, Huatulco, and Santa Rosa. Trees 2–6 m in height were cut down, and tissues were separated and dried at 60°C for 5 d. Weights were assessed with a Pesola Medio-line spring scale (1,000 g). Leaves were counted on trees of 0.7–6 m in height at the three sites in June–July 2008, 2009, or 2010 (Chamela: *N* = 26; Huatulco: *N* = 31; Santa Rosa: *N* = 24). Average leaf area was assessed for each tree by measuring the length and width of 30 haphazardly chosen leaves and converting to leaf area using the coefficients of the linear relationship, established separately for 30 leaves from each site, between leaf length×width and total area. Total leaf area for each tree was then estimated as the product of the mean individual leaf area and the total number of leaves.

For tree nutrients, samples were collected from six trees, 2 to 6 m in height, at each site in June–July 2009, following protocols described above for carbohydrate-sample collection. All samples were homogenized prior to analysis by milling. To estimate percent nitrogen and phosphorus, 50±5 mg of homogenized plant sample were Kjeldahl-digested and analyzed on a continuous flow autoanalyzer (Alpkem Flow Solution IV).

### Insurance Model of Carbon Trading

#### Tree’s perspective

Tree leaf-area growth during the early rainy season is given by:
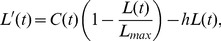
(1)where 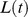 is the plant's total leaf area, 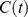 is the amount of carbon stores within the tree, 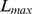 is the maximum leaf area that the tree can attain within the rainy season, and 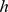 is a constant herbivory rate. We assume that the rate of photosynthesis is equal to tree leaf area, and a fraction 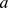 of the photosynthate is directed to consumption by scale insects. The remaining fraction 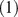 is the carbon 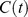, used for maintenance and new leaf production. The dynamics of the carbon pool are given by:
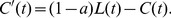
(2)We assume that the tree's reproductive output, 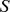, is proportional to its carbon.

#### Ant colony's perspective

The ant colony grows on photosynthate from the tree. The ant colony allocates a fraction *u* of production to new workers, and the remainder 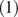 makes reproductives, 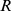. We assume that each worker consumes constant photosynthate for its own energy needs, so the maintenance cost of existing workers increases as the number of workers (colony size, 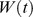) increases. The colony-size dynamics are determined by:
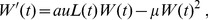
(3)where 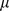 is a parameter denoting the maintenance cost of existing workers.

To determine how the allocation strategies of the tree (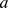) and the ant colony (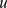) evolve to maximize their reproductive output, we make a simplifying assumption by solving for equilibrium. We obtain for the equilibrium values of our state variables:
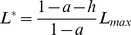
(4)

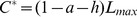
(5)

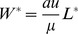
(6)The tree's 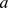 and the ant colony's 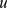 are their evolutionary strategies. For simplicity, we assume that both strategies evolve to maximize reproductive output within a rainy season.

#### Ant defense against complete defoliation

Ants protect trees from herbivory, and herbivory can produce severe defoliation. We assume that whenever the tree is defoliated, which happens with probability 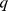, it immediately starts replacing its leaves, which requires a constant time *τ* during which the tree cannot photosynthesize. Ant colonies decrease the probability of complete defoliation proportionally to ant density 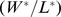 because larger trees need more ants than smaller trees to defend themselves equivalently. Thus, the expected growing time for both the plant and the ant colony, 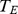, is equal to 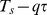, where 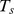 is the duration of the rainy season. We assume that the expected total reproductive output of the tree, 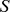, is a monotonic function of the size of its carbon pool multiplied by the time available for photosynthesis:
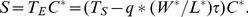
(7)Similarly, the reproductive output of the ant colony, 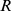, depends on the rate at which reproductives are produced multiplied by how long the colony receives carbon from the tree:
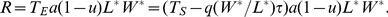
(8)


We assume that 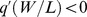, a larger colony, provides more protection against defoliation, but 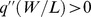, the marginal gain in protection, diminishes as the colony grows. The first-order conditions for the optimal allocation to ants by the tree, 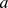, and the optimal reproductive allocation by the ants, 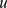, are thus:
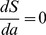
(9)

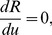
(10)which, together with second-order conditions 
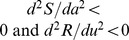
, define the ESS of the tree and the ant colony.

#### Change in the mutualism outcome across an environmental gradient

As the rainy season duration, 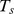, changes, we assume a particular functional form for how the probability of defoliation, 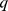, changes with the size of the colony hosted by the tree:
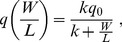
(11)where 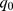 is the baseline probability of defoliation, and 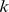 is a shape parameter that signifies what colony size is required to reduce 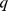 to one half of 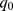. We assume that the probability of defoliation decreases with ant density according to a hyperbolic decay function. To find the ESS 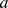 and 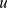, we substitute (11) into equations (9) and (10), solve the resulting equations simultaneously for 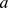 and 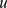, and verify which of the solutions satisfy the second-order conditions. We also investigated analytically and numerically how changes in 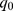 with 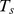 affected the direction of our ESS results (see Figure S10).

### Data Analyses

All analyses were conducted using JMP 9.0.0 or R 2.14.2 using general linear models and adjusted 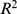 values. Raw data were deposited in the Dryad Digital Repository [57]; for a summary of the data employed, see Table 1.

## Supporting Information

Figure S1 **Detailed precipitation data for sites Chamela, Huatulco, and Santa Rosa.** (A) Total annual precipitation (1980–2009) and (b) averaged monthly precipitation (1979–2009) for the three sites highlighted in Figure 1. White represents Chamela, Mexico, gray represents Huatulco, Mexico, and black represents Santa Rosa, Costa Rica. The duration of the growing season for dry-season deciduous trees, such as *Cordia alliodora*, is determined by the number of months with precipitation ≥100 mm. Data were taken from weather stations at the biological stations associated to Chamela and Santa Rosa, and from incomplete datasets for three weather stations in towns ∼23–41 km from Huatulco.
(TIF)

Figure S2 **Time lag between host–plant colonization and a defensive ant colony.** Ants defend against herbivores only during the rainy season when the tree has leaves. For trees to replace their ant colonies between rainy seasons, ant colonies would need to grow large enough to be active defenders within 1 year. We planted 144 two-year-old, greenhouse-grown, ant-free seedlings in the field in 2009 and followed them for the next 3 years (2010–2012), checking every 6 months whether plants had a defending colony. At the end of 3 years, 89 of the plants (62%) had survived and of these 28 (31%) had defending colonies. For these 28 plants, it took significantly longer than 12 months for colonies to become defensive (*Z* = −5.37, *p*<0.0001).
(TIF)

Figure S3 (A) Natural abundance of ∂^15^N in plant tissue (domatia), scale insects, and *A. pittieri* ants (head and alitrunk) at a drier site (Chamela) and a wetter site (Santa Rosa). Different letters indicate *p*<0.05 by ANOVA and Tukey HSD. (B) Percent carbon in *A. pittieri* ants (head and alitrunk) at a drier site (Chamela) and a wetter site (Santa Rosa). (C) Percent nitrogen in *A. pittieri* ants (head and alitrunk) at a drier site (Chamela) and a wetter site (Santa Rosa). In all plots, boxes indicate the median (bold line) and quartiles; points are outliers.
(TIF)

Figure S4 **Relationship between leaf herbivory and the latency of ant response to a standardized disturbance to the host tree.** Leaf herbivory was measured as an index based on the standing percentage of leaf area eaten; the standardized disturbance was caused by hitting the tree with a hammer. The herbivory index was calculated by assigning leaves to the ordinal categories 0–5 (corresponding to percentages of missing leaf area: 0 = 0%, 1 = >1–6%, 2 = >6–12%, 3 = 12–25%, 4 = 25–50%, 5 = >50–100%), multiplying the number of leaves in each category by the category value, taking the sum for all categories, and dividing by the total number of leaves.
(TIF)

Figure S5 **Behavioral assays of ant defensive efficacy.** (A) Ant patrolling intensity, measured as the number of ants on leaves visible in a 1-min scan and standardized by tree size. (B) Latency of ant response to disturbance of the tree by hitting it with a hammer. Different lowercase letters indicate significant differences (Tukey HSD tests, *p*<0.0001).
(TIF)

Figure S6 **Total nonstructural carbohydrate pools at a drier and a wetter site.** Storage of total NSCs in (A) main stems and (B) twigs of *C. alliodora* trees at three seasonal stages: early rainy season, June–July 2008 and 2010; late rainy or early dry season, October 2009; and late dry season, April 2009. White bars represent the drier site (Chamela); black bars represent the wetter site (Santa Rosa). For each bar, starch is the bottom (open), free glucose is the middle (diagonal lines), and sucrose is the top (vertical lines). Error bars represent SE of total NSCs; values are based on plant dry weights. Note the difference in scale between (A) and (B). For full statistical results, see Tables S6 and S7.
(TIF)

Figure S7 **Tree-growth allometries at three representative dry-forest sites.** (A) Dry weight of all stem tissue (main stem and twigs) by tree basal diameter. (B) Dry weight of leaves by dry weight of all stem tissue. (C) Total leaf area, calculated as the total number of leaves multiplied by the average leaf area of 30 representative leaves, by tree basal diameter. The interaction between site and the independent continuous variable was significant only in (C) (full ANCOVA model, *F*_5,86_ = 39.83, *p*<0.0001; site, *p* = 0.07; basal diameter, *p*<0.0001; site×basal diameter, *p*<0.02), driven by the difference in least-squares-means differences between Chamela and Santa Rosa. However, in direct least-squares-means comparisons, the slope between leaf area and basal diameter at Chamela (m = 1.77±0.19) was not significantly greater than the slope at Santa Rosa (m = 1.49±0.32; Tukey HSD N.S.). Lines represent log–log transformations of both variables. Open circles and solid lines represent trees from the driest site, Chamela; gray diamonds and dashed lines represent trees from the dry site of intermediate latitude, Huatulco; black triangles and double-dashed lines represent trees from the wettest site, Santa Rosa.
(TIF)

Figure S8 **Nitrogen∶phosphorus ratios in tree twigs, stems, roots, and leaves.** Mean ± SE of N∶P ratios in different tissues of *C. alliodora* juvenile trees at the three sites (driest, Chamela; dry site of intermediate latitude, Huatulco; and wettest, Santa Rosa), collected in the early rainy season of 2008 (roots only) and 2009. Different lowercase letters indicate significant differences by ANOVA (*F*_2,15_ = 4.77, *p*<0.03) and Tukey HSD (*p*<0.05).
(TIF)

Figure S9 **Background herbivory in ant-exclusion experiments.** Percent leaf area eaten over 3 weeks (mean + SE) when ants were experimentally excluded from leaves.
(TIF)

Figure S10 **Changes in background herbivory in the insurance model.** The change in ESS tree allocation and ant defense when *q_0_* (baseline probability of defoliation) is a linearly increasing function of the growing-season length. The figure shows that for a large enough increase in the defoliation probability with growing-season length, a positive relationship between mutualism strength and growing-season length is possible. We obtained an analytic expression for the threshold rate of increase of *q_0_* with *T_s_*, but it is so cumbersome that we instead illustrate this feature of the model numerically. Here, we assumed that *q_0_* = 0.1+θ(*T_s_*-5), with θ = 0.03, 0.02, and, 0.01 for the dashed-dotted, dashed, and solid curves, respectively. Other parameter values are: *h* = 0.1, *L*_max_ = 10, *τ* = 5, *μ* = 0.01, *k* = 1.
(TIF)

Figure S11 **Changes in mutualism outcomes predicted from the chronic-herbivory model (Text S1).** The equilibrium (A) tree carbon pool, C^*^, and (B) ant colony size, W^*^, at the evolutionary-stable-strategy allocation values. The evolutionary-stable-strategy values of (C) tree carbohydrate investment in ants, *a*, and (D) ant investment in colony growth, *u*, as a function of the background herbivory *h_0_*. Parameter values are: *q* = 0.2, *L_max_* = 10, *T_max_* = 15, *τ* = 3, *μ* = 0.01, *k_h_* = 10.
(TIF)

Table S1 **The 26 Mesoamerican study sites, annual precipitation, and number of host trees examined.**
(XLS)

Table S2 **Maximum and minimum temperatures at three representative sites ± SD.**
(XLS)

Table S3 **Plant-available soil nutrients in ppm (µg/g) (mean ± SE).**
(XLS)

Table S4 **Tree growth (cm) per year in a drier site, Chamela, and a wetter site, Santa Rosa.**
(XLS)

Table S5 **Number of female alates in completely dissected colonies at three sites spanning the dry forest precipitation gradient.**
(XLS)

Table S6 **Mean percent NSCs ± SE in main stems, twigs, leaves, and roots from a drier and a wetter site in three seasons.**
(XLS)

Table S7 **General linear models of NSC concentrations in a drier site, Chamela, and a wetter site, Santa Rosa, with site, season, and their interaction as fixed effects.**
(XLS)

Text S1 **An alternate, chronic-herbivory mathematical model.**
(DOCX)

## Abbreviations

NSC: nonstructural carbohydrates
