# Supplementary figures and images for: Water Stress Strengthens Mutualism Among Ants, Trees, and Scale Insects
Source: PLoS Biol. 2013 Nov 5;11(11):e1001705. doi: 10.1371/journal.pbio.1001705 (PMC3818173; doi:10.1371/journal.pbio.1001705)

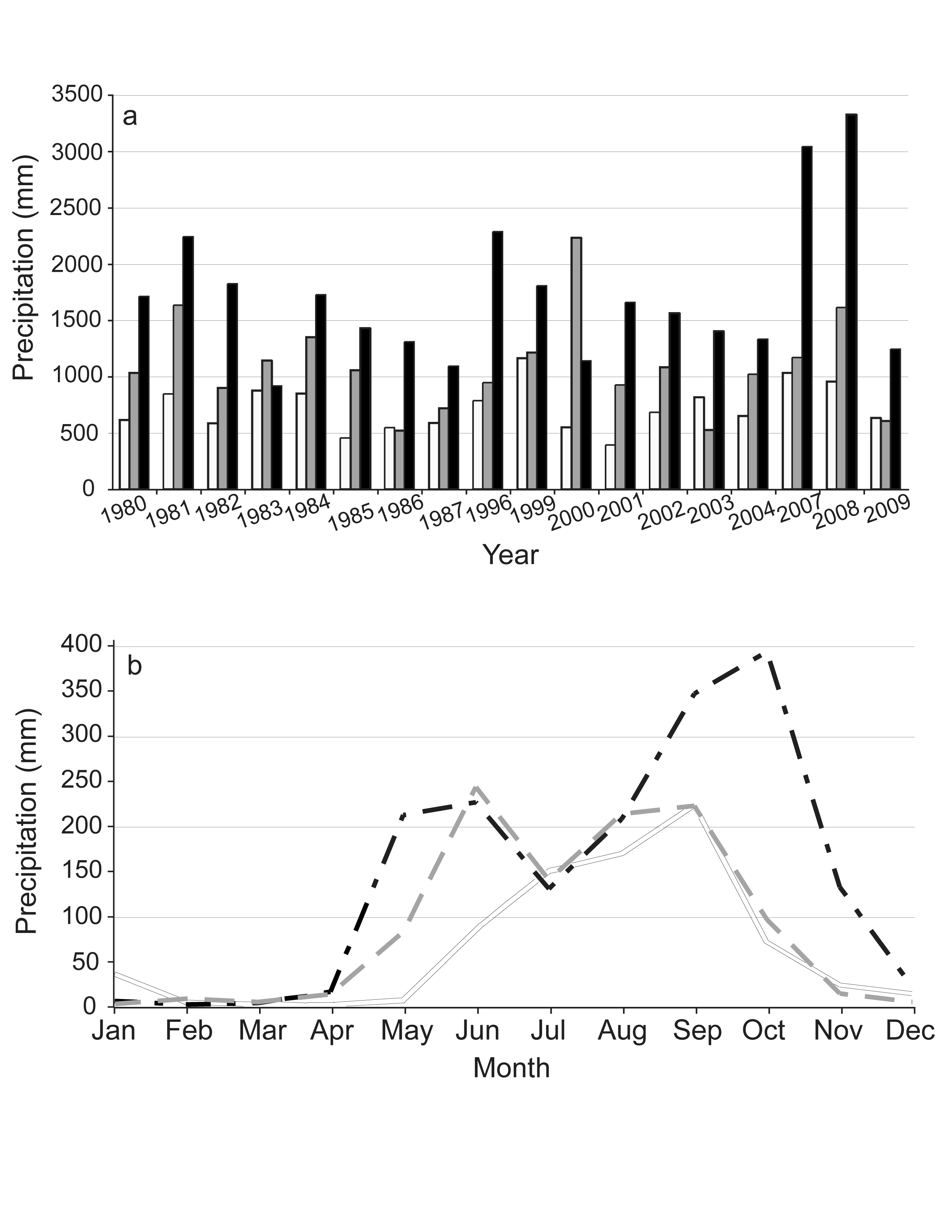

Supplement: Figure S1 — Detailed precipitation data for sites Chamela, Huatulco, and Santa Rosa. (A) Total annual precipitation (1980–2009) and (b) averaged monthly precipitation (1979–2009) for the three sites highlighted in Figure 1. White represents Chamela, Mexico, gray represents Huatulco, Mexico, and black represents Santa Rosa, Costa Rica. The duration of the growing season for dry-season deciduous trees, such as Cordia alliodora, is determined by the number of months with precipitation ≥100 mm. Data were taken from weather stations at the biological stations associated to Chamela and Santa Rosa, and from incomplete datasets for three weather stations in towns ∼23–41 km from Huatulco. (TIF) [file pbio.1001705.s001.tif]

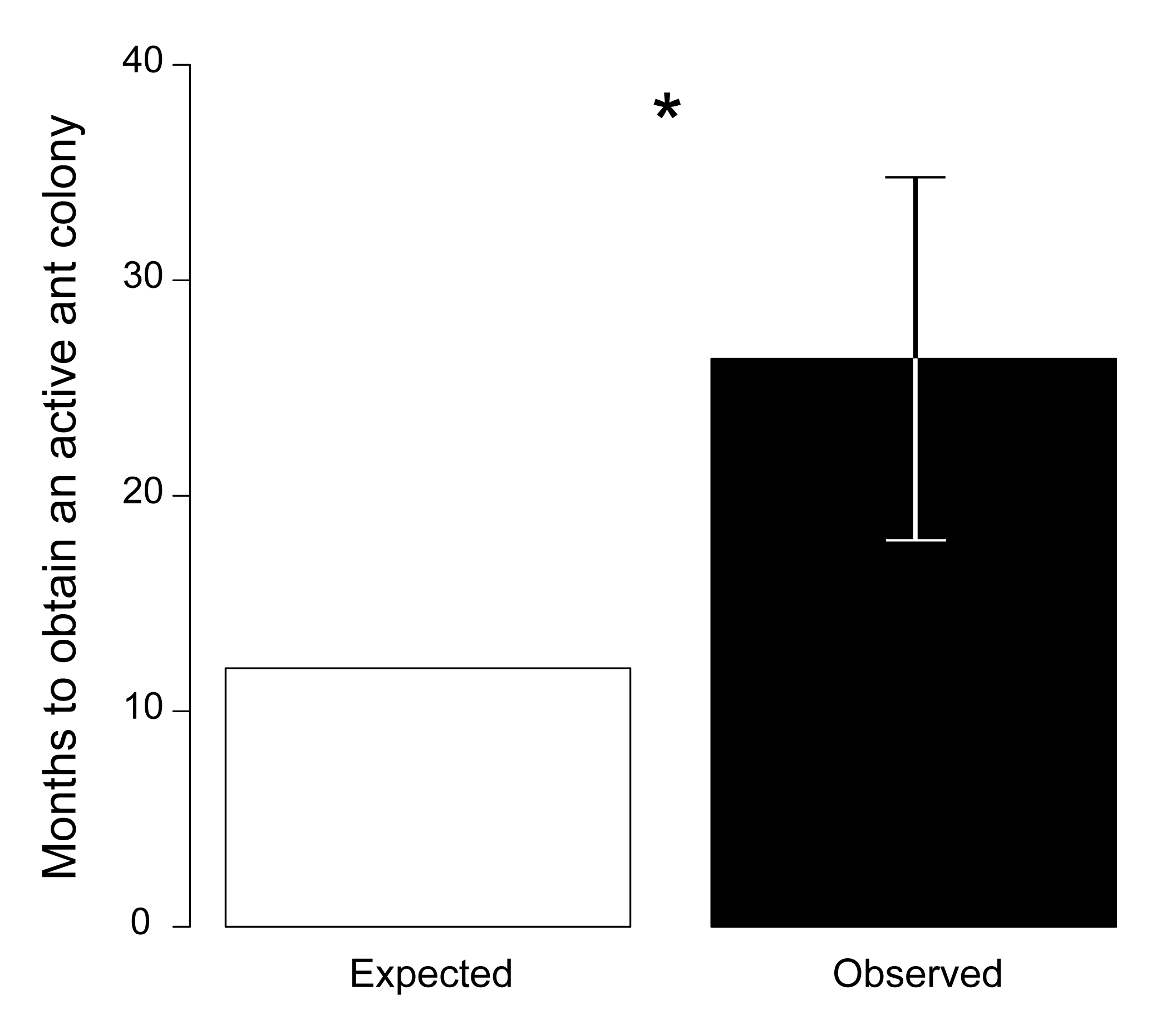

Supplement: Figure S2 — Time lag between host–plant colonization and a defensive ant colony. Ants defend against herbivores only during the rainy season when the tree has leaves. For trees to replace their ant colonies between rainy seasons, ant colonies would need to grow large enough to be active defenders within 1 year. We planted 144 two-year-old, greenhouse-grown, ant-free seedlings in the field in 2009 and followed them for the next 3 years (2010–2012), checking every 6 months whether plants had a defending colony. At the end of 3 years, 89 of the plants (62%) had survived and of these 28 (31%) had defending colonies. For these 28 plants, it took significantly longer than 12 months for colonies to become defensive (Z = −5.37, p<0.0001). (TIF) [file pbio.1001705.s002.tif]

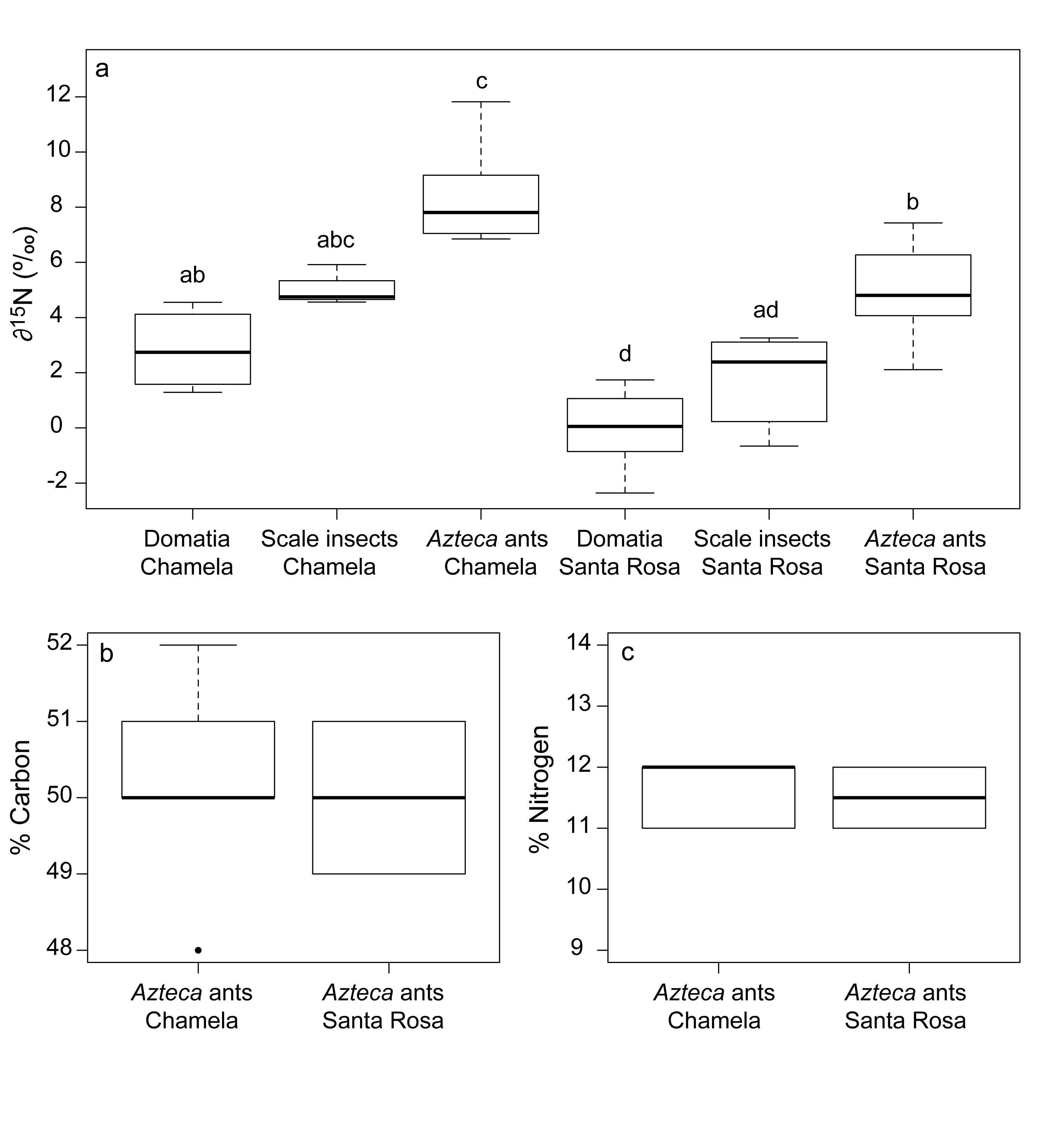

Supplement: Figure S3 — (A) Natural abundance of ∂15N in plant tissue (domatia), scale insects, and A. pittieri ants (head and alitrunk) at a drier site (Chamela) and a wetter site (Santa Rosa). Different letters indicate p<0.05 by ANOVA and Tukey HSD. (B) Percent carbon in A. pittieri ants (head and alitrunk) at a drier site (Chamela) and a wetter site (Santa Rosa). (C) Percent nitrogen in A. pittieri ants (head and alitrunk) at a drier site (Chamela) and a wetter site (Santa Rosa). In all plots, boxes indicate the median (bold line) and quartiles; points are outliers. (TIF) [file pbio.1001705.s003.tif]

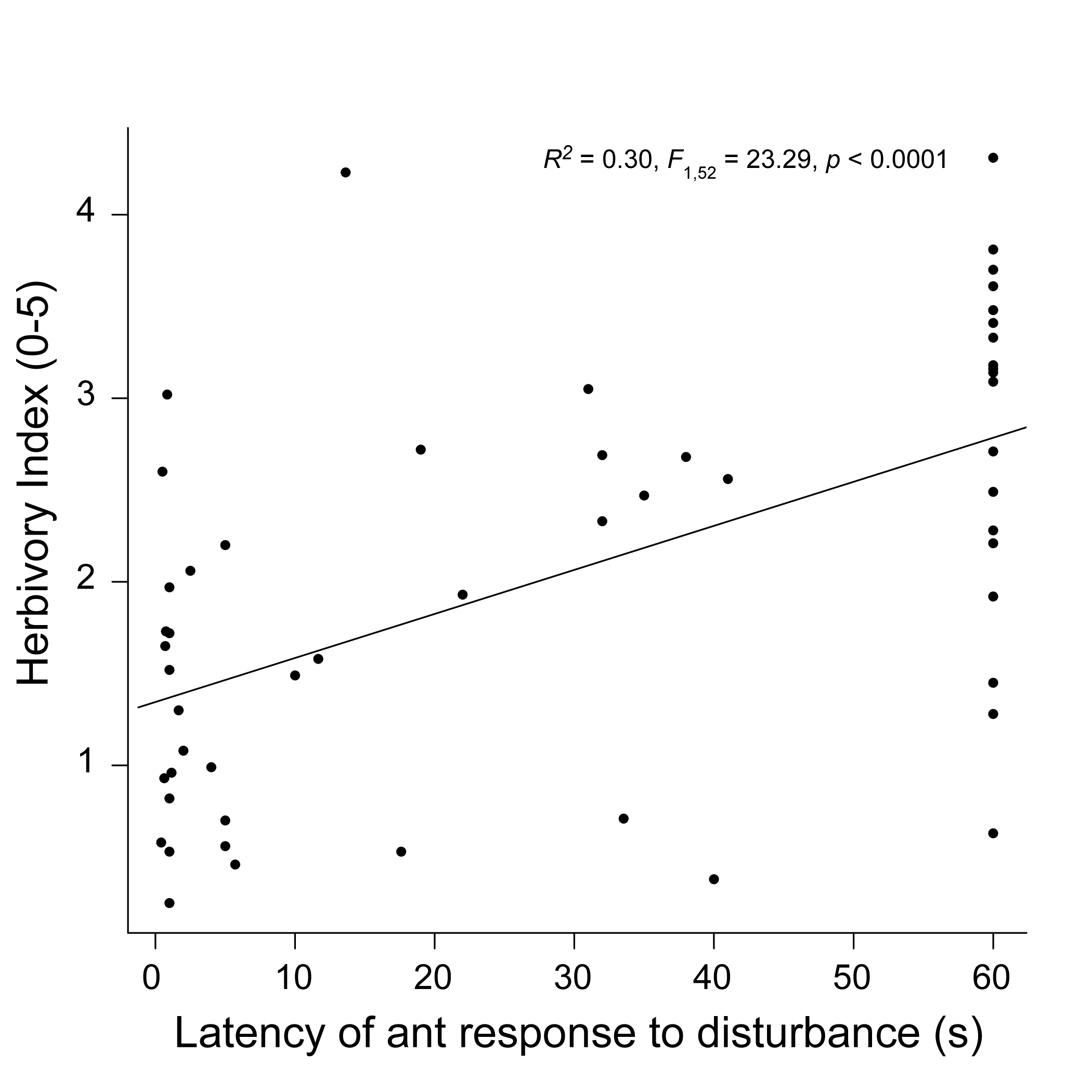

Supplement: Figure S4 — Relationship between leaf herbivory and the latency of ant response to a standardized disturbance to the host tree. Leaf herbivory was measured as an index based on the standing percentage of leaf area eaten; the standardized disturbance was caused by hitting the tree with a hammer. The herbivory index was calculated by assigning leaves to the ordinal categories 0–5 (corresponding to percentages of missing leaf area: 0 = 0%, 1 = >1–6%, 2 = >6–12%, 3 = 12–25%, 4 = 25–50%, 5 = >50–100%), multiplying the number of leaves in each category by the category value, taking the sum for all categories, and dividing by the total number of leaves. (TIF) [file pbio.1001705.s004.tif]

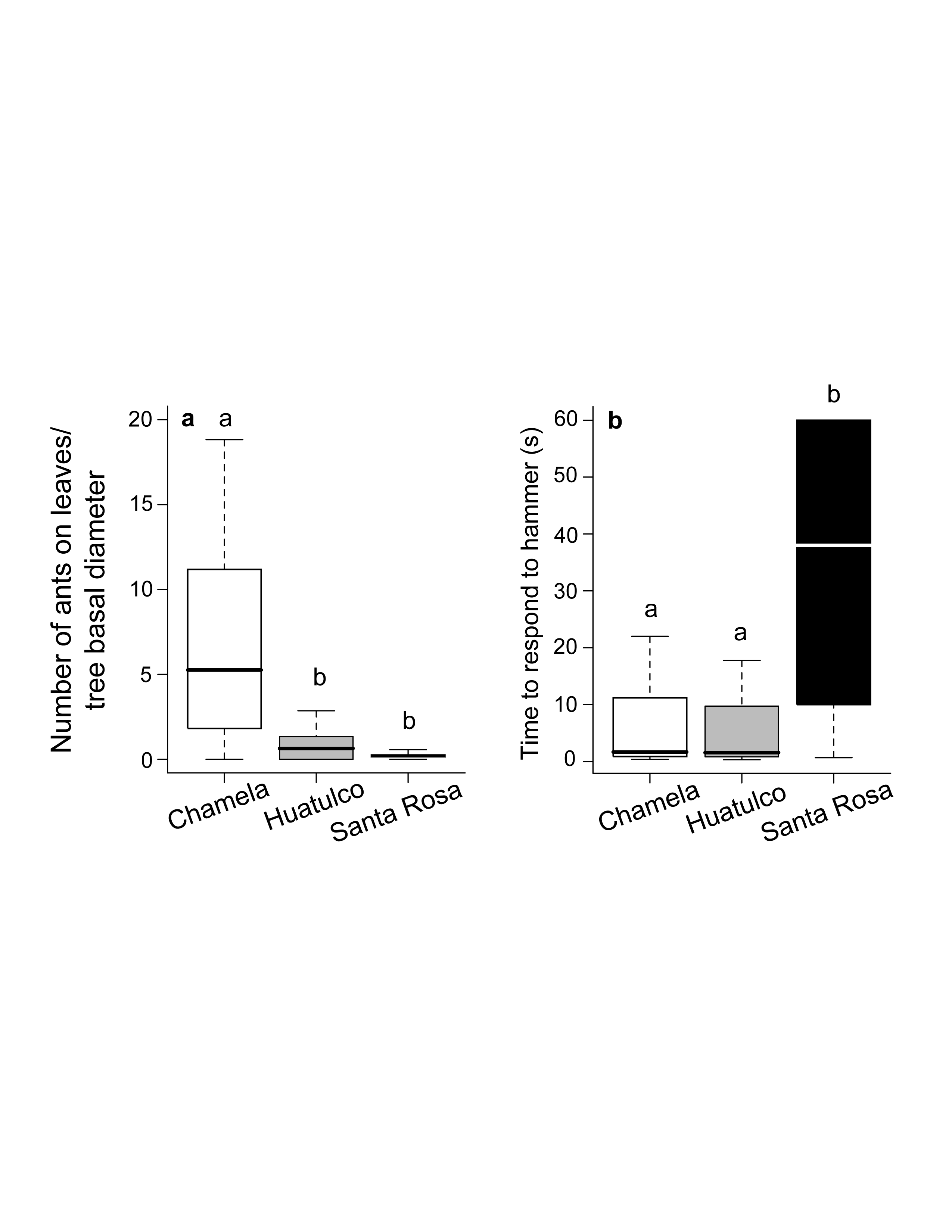

Supplement: Figure S5 — Behavioral assays of ant defensive efficacy. (A) Ant patrolling intensity, measured as the number of ants on leaves visible in a 1-min scan and standardized by tree size. (B) Latency of ant response to disturbance of the tree by hitting it with a hammer. Different lowercase letters indicate significant differences (Tukey HSD tests, p<0.0001). (TIF) [file pbio.1001705.s005.tif]

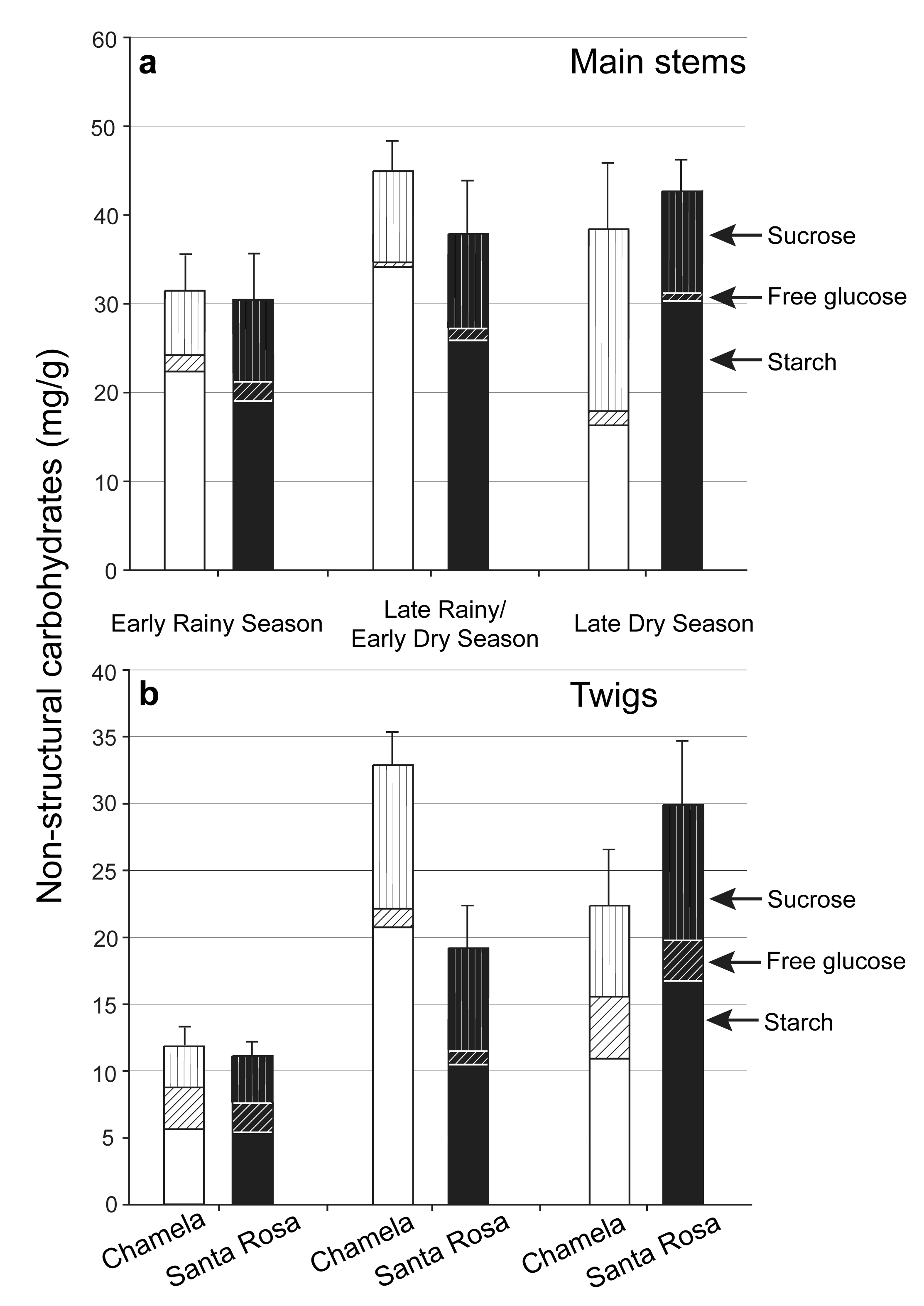

Supplement: Figure S6 — Total nonstructural carbohydrate pools at a drier and a wetter site. Storage of total NSCs in (A) main stems and (B) twigs of C. alliodora trees at three seasonal stages: early rainy season, June–July 2008 and 2010; late rainy or early dry season, October 2009; and late dry season, April 2009. White bars represent the drier site (Chamela); black bars represent the wetter site (Santa Rosa). For each bar, starch is the bottom (open), free glucose is the middle (diagonal lines), and sucrose is the top (vertical lines). Error bars represent SE of total NSCs; values are based on plant dry weights. Note the difference in scale between (A) and (B). For full statistical results, see Tables S6 and S7. (TIF) [file pbio.1001705.s006.tif]

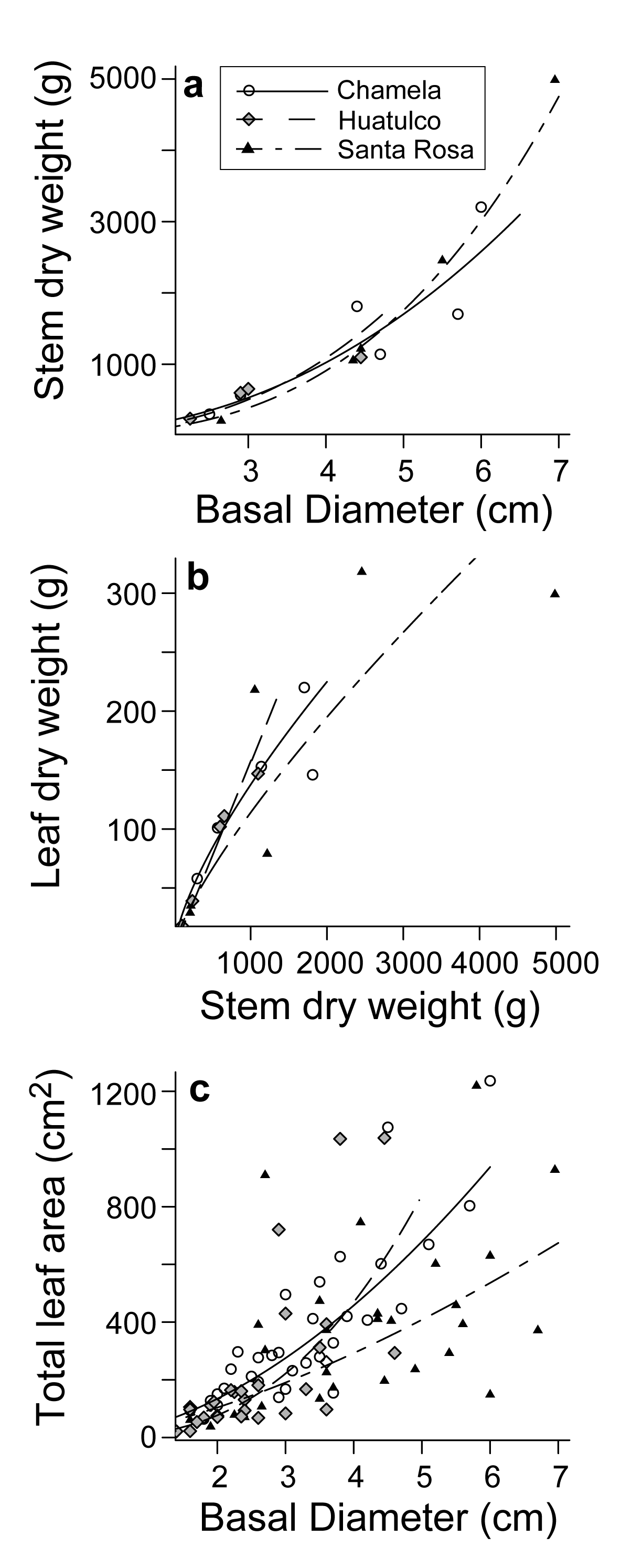

Supplement: Figure S7 — Tree-growth allometries at three representative dry-forest sites. (A) Dry weight of all stem tissue (main stem and twigs) by tree basal diameter. (B) Dry weight of leaves by dry weight of all stem tissue. (C) Total leaf area, calculated as the total number of leaves multiplied by the average leaf area of 30 representative leaves, by tree basal diameter. The interaction between site and the independent continuous variable was significant only in (C) (full ANCOVA model, F 5,86 = 39.83, p<0.0001; site, p = 0.07; basal diameter, p<0.0001; site×basal diameter, p<0.02), driven by the difference in least-squares-means differences between Chamela and Santa Rosa. However, in direct least-squares-means comparisons, the slope between leaf area and basal diameter at Chamela (m = 1.77±0.19) was not significantly greater than the slope at Santa Rosa (m = 1.49±0.32; Tukey HSD N.S.). Lines represent log–log transformations of both variables. Open circles and solid lines represent trees from the driest site, Chamela; gray diamonds and dashed lines represent trees from the dry site of intermediate latitude, Huatulco; black triangles and double-dashed lines represent trees from the wettest site, Santa Rosa. (TIF) [file pbio.1001705.s007.tif]

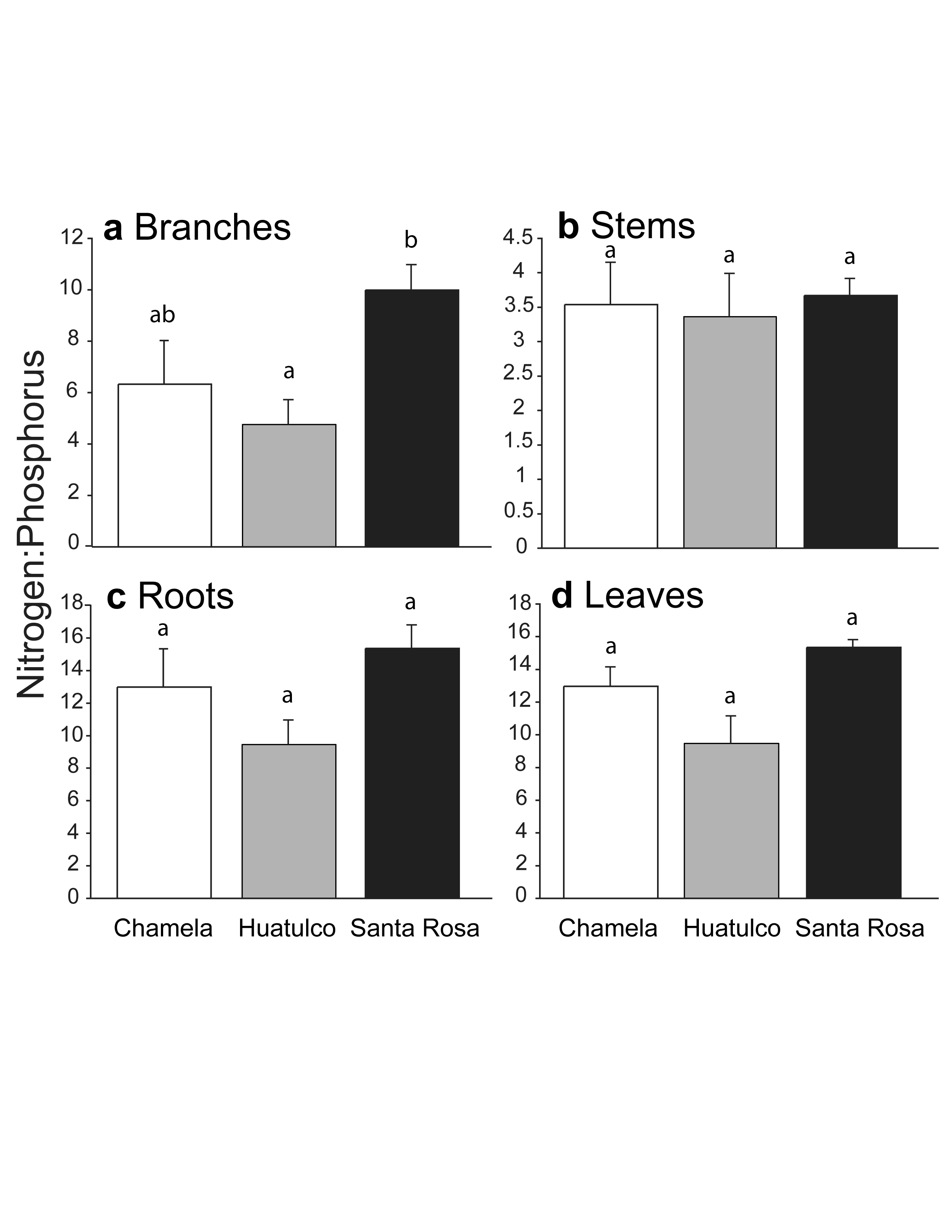

Supplement: Figure S8 — Nitrogen∶phosphorus ratios in tree twigs, stems, roots, and leaves. Mean ± SE of N∶P ratios in different tissues of C. alliodora juvenile trees at the three sites (driest, Chamela; dry site of intermediate latitude, Huatulco; and wettest, Santa Rosa), collected in the early rainy season of 2008 (roots only) and 2009. Different lowercase letters indicate significant differences by ANOVA (F 2,15 = 4.77, p<0.03) and Tukey HSD (p<0.05). (TIF) [file pbio.1001705.s008.tif]

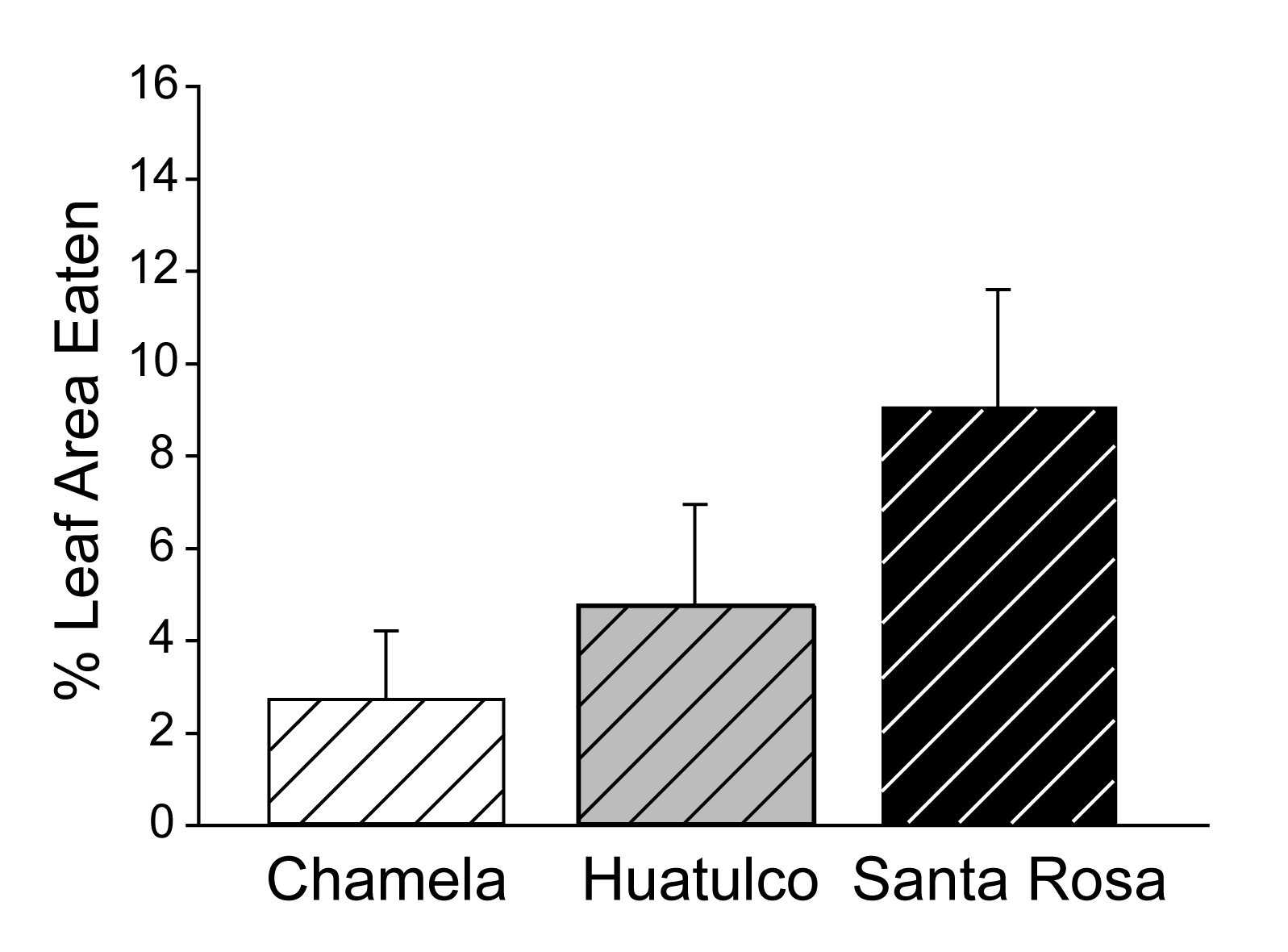

Supplement: Figure S9 — Background herbivory in ant-exclusion experiments. Percent leaf area eaten over 3 weeks (mean + SE) when ants were experimentally excluded from leaves. (TIF) [file pbio.1001705.s009.tif]

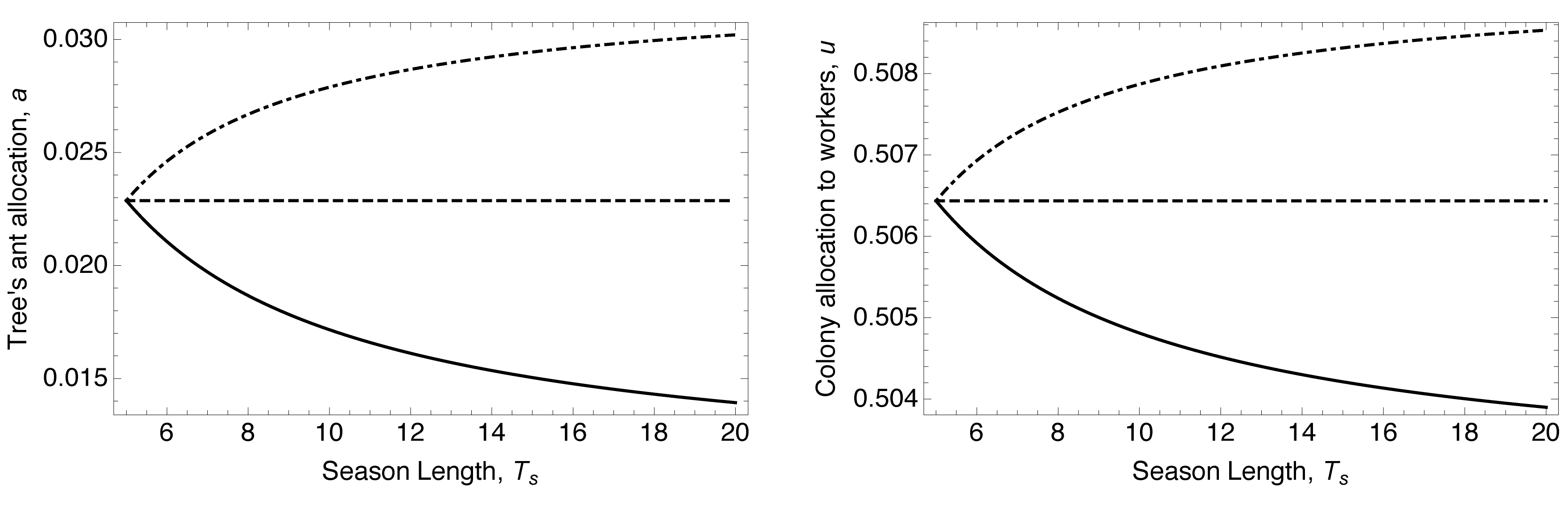

Supplement: Figure S10 — Changes in background herbivory in the insurance model. The change in ESS tree allocation and ant defense when q0 (baseline probability of defoliation) is a linearly increasing function of the growing-season length. The figure shows that for a large enough increase in the defoliation probability with growing-season length, a positive relationship between mutualism strength and growing-season length is possible. We obtained an analytic expression for the threshold rate of increase of q0 with Ts, but it is so cumbersome that we instead illustrate this feature of the model numerically. Here, we assumed that q0 = 0.1+θ(Ts-5), with θ = 0.03, 0.02, and, 0.01 for the dashed-dotted, dashed, and solid curves, respectively. Other parameter values are: h = 0.1, L max = 10, τ = 5, μ = 0.01, k = 1. (TIF) [file pbio.1001705.s010.tif]

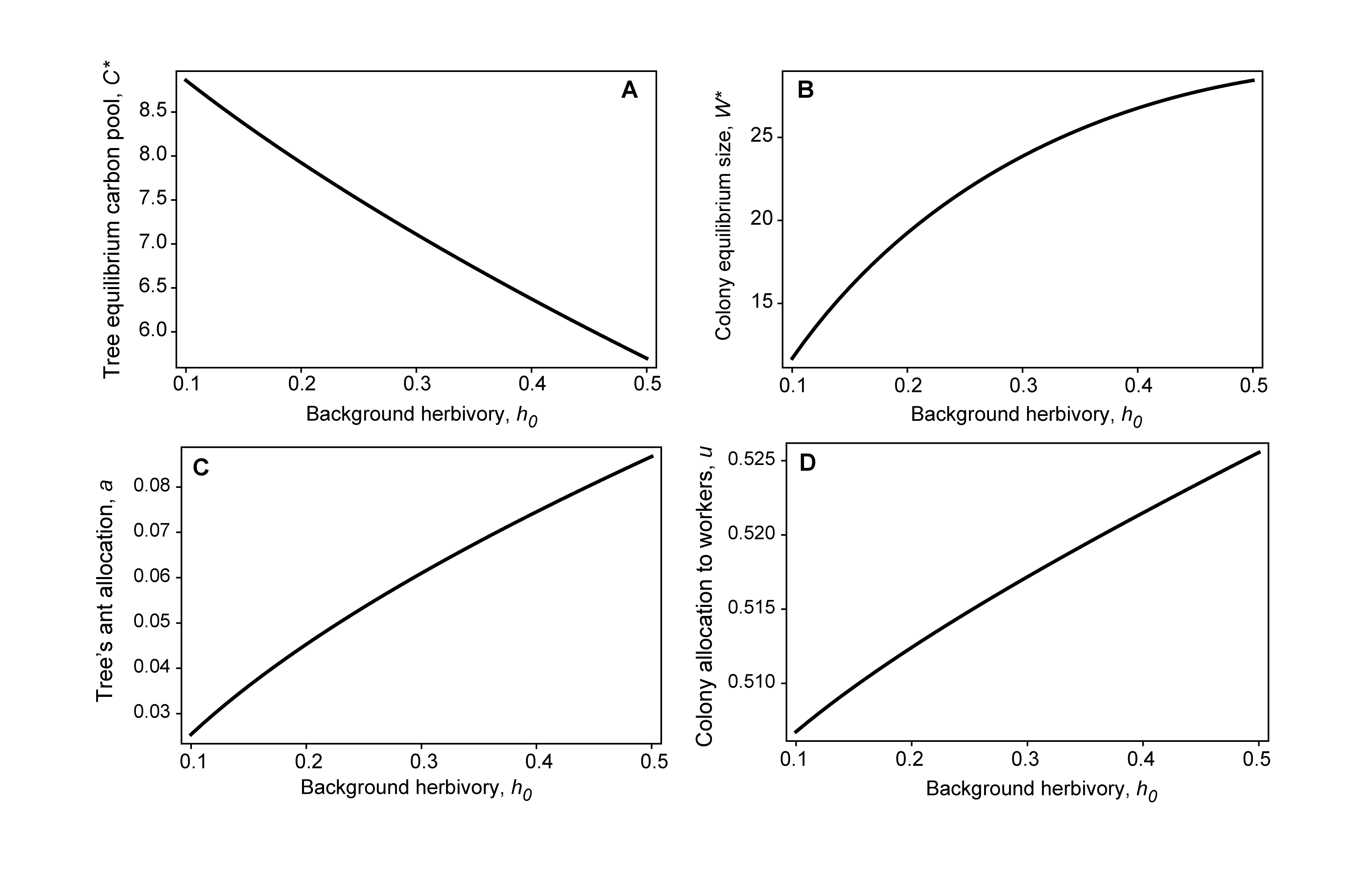

Supplement: Figure S11 — Changes in mutualism outcomes predicted from the chronic-herbivory model (Text S1). The equilibrium (A) tree carbon pool, C*, and (B) ant colony size, W*, at the evolutionary-stable-strategy allocation values. The evolutionary-stable-strategy values of (C) tree carbohydrate investment in ants, a, and (D) ant investment in colony growth, u, as a function of the background herbivory h0. Parameter values are: q = 0.2, Lmax = 10, Tmax = 15, τ = 3, μ = 0.01, kh = 10. (TIF) [file pbio.1001705.s011.tif]
