# Supplementary material for: Water Stress Strengthens Mutualism Among Ants, Trees, and Scale Insects
Source: PLoS Biol. 2013 Nov 5;11(11):e1001705. doi: 10.1371/journal.pbio.1001705 (PMC3818173; doi:10.1371/journal.pbio.1001705)
Supplement: Text S1 — An alternate, chronic-herbivory mathematical model. (DOCX) [file pbio.1001705.s019.docx]

**Text S1 - Chronic herbivory**

Here we consider an alternate version of our model to ask whether ant defense against chronic herbivory throughout the growing season could drive the observed variation in the mutualism outcome. The dynamics of the tree’s and the ant colony’s growth are identical to Model 1, except that the constant background herbivory rate *h* is now replaced by a realized herbivory rate *h(W/L)* which is a function of the ant density on the tree:

| $L©(t)=C(t)(1-\frac{L(t)}{L_{\max}})-h\left(W(t)/L(t)\right)L(t)$ | (S1) |
| --- | --- |
| $C©(t)=(1-a)L(t)-C(t) .$ | (S2) |

| $W©(t)=\left(auL(t)-\mu W(t)\right)W(t) .$ | (S3) |
| --- | --- |

Thus, the main effect of ant defence in this model is to lower the rate at which leaves are being removed from the tree continuously throughout the growing season, given by the last term in equation (S1). Hence, this version of the model represents sustained, chronic herbivory.

We further assume that ant colony density reduces the realized herbivory according to the function

| $h(\frac{W}{L})=\frac{k_{h}h_{0}}{k_{h}+\frac{W}{L}} ,$ | (S4) |
| --- | --- |

where *h_0_* is the background herbivory in the absence of ants, and *k_h_* is a shape parameter. Substituting equation (S4) into (S1) and solving (S1), (S2), and (S3) simultaneously, we obtain for the equilibrium of the within-season dynamics:

| $L^{*}=\frac{\text{k}_{\text{h}}L_{\max}\mu(1-a-h_{0})+ua(1-a)L_{\max}}{(1-a)(ua+k_{h}\mu)}$ | (S5) |
| --- | --- |
| $C^{*}=\frac{uah_{0}\text{L}_{\text{max }}}{ua+k_{h}\mu}$ | (S6) |

| $W^{*}=uaL_{\max}\left(\frac{1}{\mu}-\frac{h_{0}k_{h}}{(1-a)(ua+k_{h}\mu)}\right) .$ | (S7) |
| --- | --- |

The expected reproduction of the tree and the ant colony is also identical to Model 1, except now the expected length in which the tree and the ant colony can reproduce is a constant, instead of depending on the ant density on the tree.

| $S=(T_{S}-q\tau)C^{*}$ | (S8) |
| --- | --- |
| $R=(T_{s}-q\tau)a(1-u)L^{*}W^{*}$ | (S9) |

Again, we assume that the tree’s ant allocation (*a*) and the ant’s allocation to workers (*u*) evolve to maximize *S* and *R*, respectively. We solve numerically for the pair of tree and ant strategies that satisfy the first and second order ESS conditions given in the main text, and ask how this equilibrium changes with changing environmental parameters.

Our first result is that the optimal strategies for both the tree and the ant colony do not change with the length of the growing season if the background herbivory rate *h_0_* stays constant. This can be immediately observed in equations (S8) and (S9): since the equilibrium of the growth dynamics (given by (S5-7)) does not depend on *T_s_*, changing the season length only scales the reproductive output of the tree and the colony, without changing the location of their maxima. Hence, season length by itself has no direct effect on the mutualism outcome in this version of the model.

However, other environmental variables can covary with the season length. In particular, background herbivory is higher in Santa Rosa, than in Chamela, so we can expect a positive relation between length of the growing season and the background herbivory rate *h_0_*. Figure S11 depicts the effect of increasing herbivory on the tree and ant strategies, and the equilibrium carbon pool of the tree and the colony size. Panels C and D, show that both the tree’s allocation to ants and the ant colony’s allocation to workers increases with *h_0_*. The intuitive explanation for these patterns is that as herbivory increases, the value for the tree of investing in defense increases. Similarly, with increased herbivory, the ant colony has more to gain from producing more workers and thereby preventing the photosynthesis rate from being reduced by herbivory. As a result of these two effects, the tree’s equilibrium carbon pool size goes down with increased herbivory, as seen in Panel A, while the ant colony’s size increases, as seen in Panel B. These patterns are opposite of what we observe in our data, which suggests that protection against chronic herbivory throughout the growing season is unlikely to explain the variation in the mutualistic outcome. We confirmed this intuition using more complex models that took into account the within-season dynamics of tree and ant colony growth, as well as the optimal timing of tree reproduction (results not shown).
